# Supplementary material for: miR-181a initiates and perpetuates oncogenic transformation through the regulation of innate immune signaling
Source: Nat Commun. 2020 Jun 26;11:3231. doi: 10.1038/s41467-020-17030-w (PMC7320168; doi:10.1038/s41467-020-17030-w)
Supplement: Supplementary file 1 — Supplementary Information [file 41467_2020_17030_MOESM1_ESM.pdf]

Supplementary Information

miR-181a initiates and perpetuates oncogenic transformation through the regulation of innate immune signaling

Knarr et al.

Supplementary Figure 1

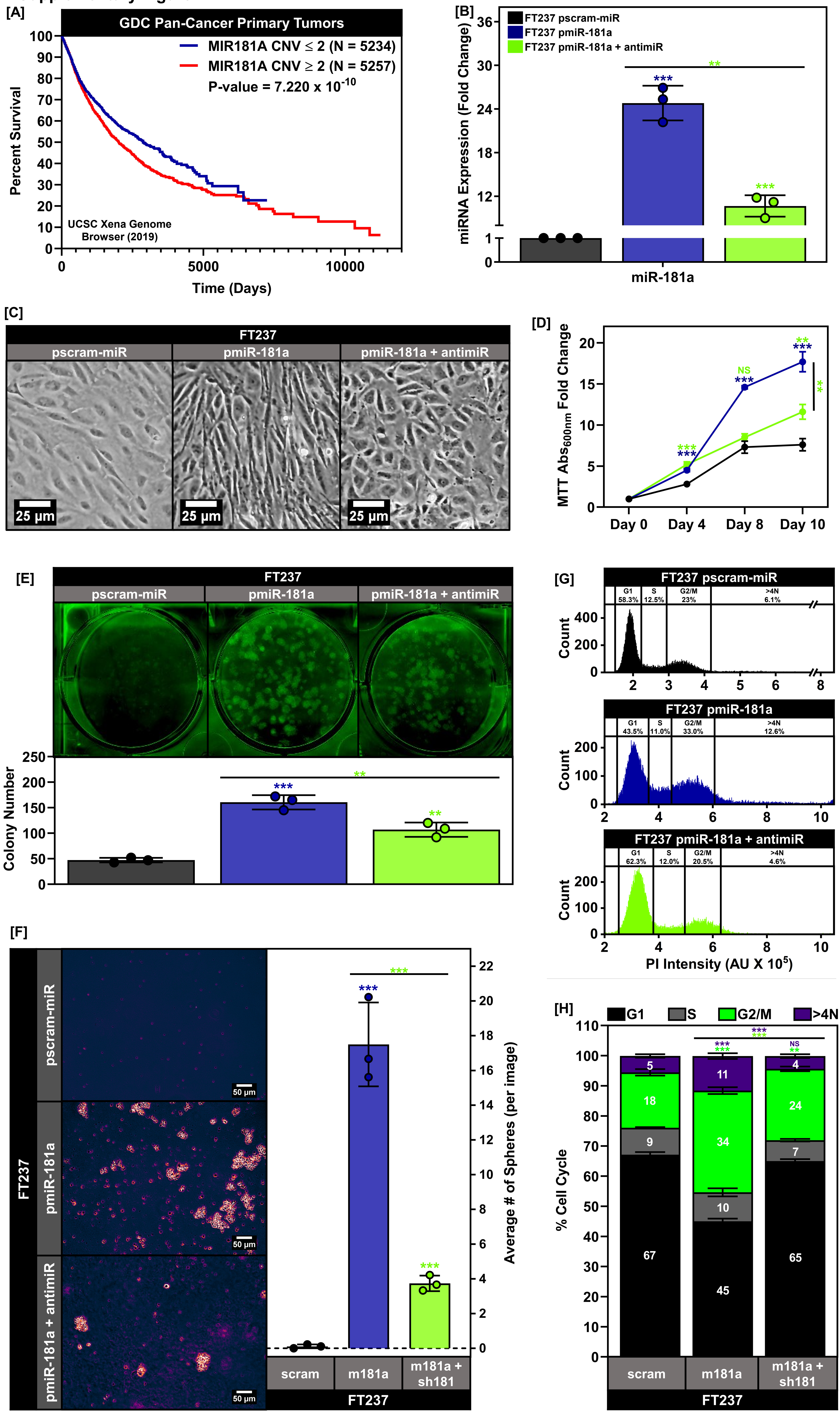

Supplementary Figure 1: Addition of miR-181a antagomiR inhibits the in vitro transformation phenotype of miR-181a overexpression in FTSECs

A) Pan-Cancer Analysis through the use of the UCSC Xena Genome Browser showed that patients with primary tumors which harbored amplification of miR-181a succumb to their disease significantly faster. The log-rank test was used for statistical analysis of survival curves. P-value shown is log-rank adjusted. B) Graph showing miR-181a expression levels for the FT237 pscram-miR, pmiR-181a, and pmiR-181a + antimiR cell lines. C) Phase contrast micrographs showing loss of contact inhibition in the FT pmiR-181a vs pscram-miR or pmiR-181a + antimiR cells. All cells were plated at the same time at equal density and allowed to grow for 10 days. D) Graph showing cell viability for FT237 pscram-miR, pmiR-181a, and pmiR-181a + antimiR cells. Significance values are color coded to match the corresponding FT237 cell line. E) Colony formation assay showing survival and colony formation for the FT237 pscram-miR, pmiR-181a, and pmiR-181a + antimiR cells with quantification (below). Colonies were stained with CellTag 700 at 10 days. Dashed green lines denote the culture plate well boundaries. F) Micrographs showing anchorage independent growth of FT237 pscram-miR, pmiR-181a, and pmiR-181a + antimiR cells with quantification (right). G) Representative graphs of cell cycle profiles for the FT237 pscram-miR, pmiR-181a, and pmiR-181a + antimiR cells. H) Bar graph of the % cell cycle sub-populations for FT237 pscram-miR, pmiR-181a, and pmiR-181a + antimiR cells. The cell cycle data for FT237 pscram-miR and pmiR-181a cells is the same as shown in Figure 1G. All data are representative of N = 3 independent experiments unless otherwise stated. The measure of center for the error bars is given as the mean value unless otherwise stated. The statistical test used for data analysis is the two-sided Student’s t-test unless otherwise stated. Error bars indicate ±standard deviation unless otherwise stated. * = $p<0.05$, ** = $p<0.005$, *** = $p<0.0005$.

Supplementary Figure 2

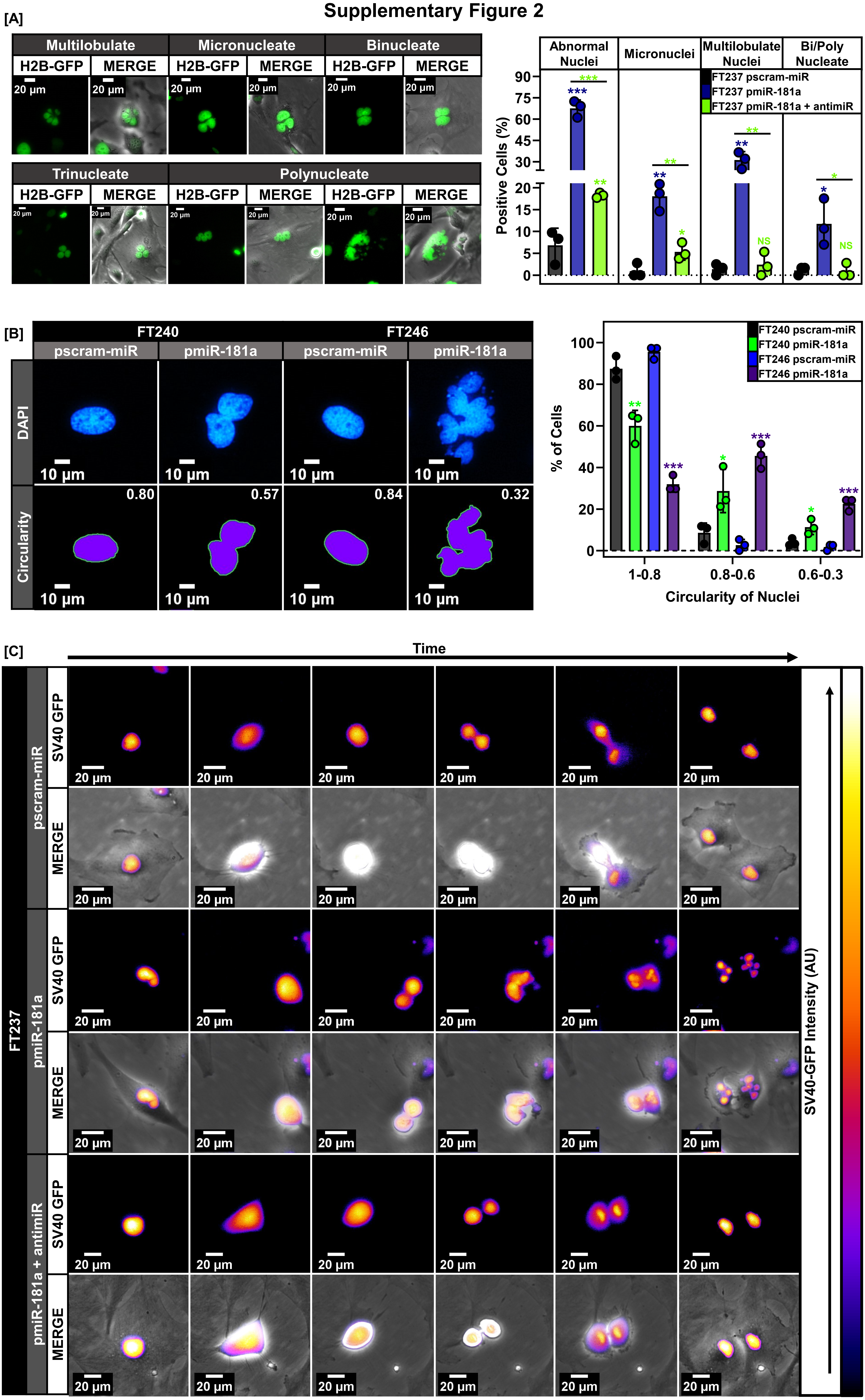

Supplementary Figure 2: miR-181a overexpression causes nuclear defects in FT237, FT240, and FT246 cells

A) Representative H2B-GFP and H2B-GFP/phase contrast merged micrographs of the types of nuclear defects seen in the FT237 pmiR-181a cells (left) and quantification of the defects (right). B) Immunofluorescence micrographs of representative DAPI stained nuclei (top), matched circularity masks with circularity value displayed in upper right hand corner (bottom), and graph showing the circularity distribution (right) from FT240 and FT246 pscram-miR and pmiR-181a cells. C) Time-lapse of SV40-GFP and SV40-GFP/phase contrast merged immunofluorescence images showing typical cell division along with parent and daughter nuclei in FT237 pscram-miR, pmiR-181a, and pmiR-181a + antimiR cells. All data are representative of N = 3 independent experiments unless otherwise stated. The measure of center for the error bars is given as the mean value unless otherwise stated. The statistical test used for data analysis is the two-sided Student's t-test unless otherwise stated. Error bars indicate ±standard deviation unless otherwise stated. * = $p<0.05$, ** = $p<0.005$, *** = $p<0.0005$.

Supplementary Figure 3

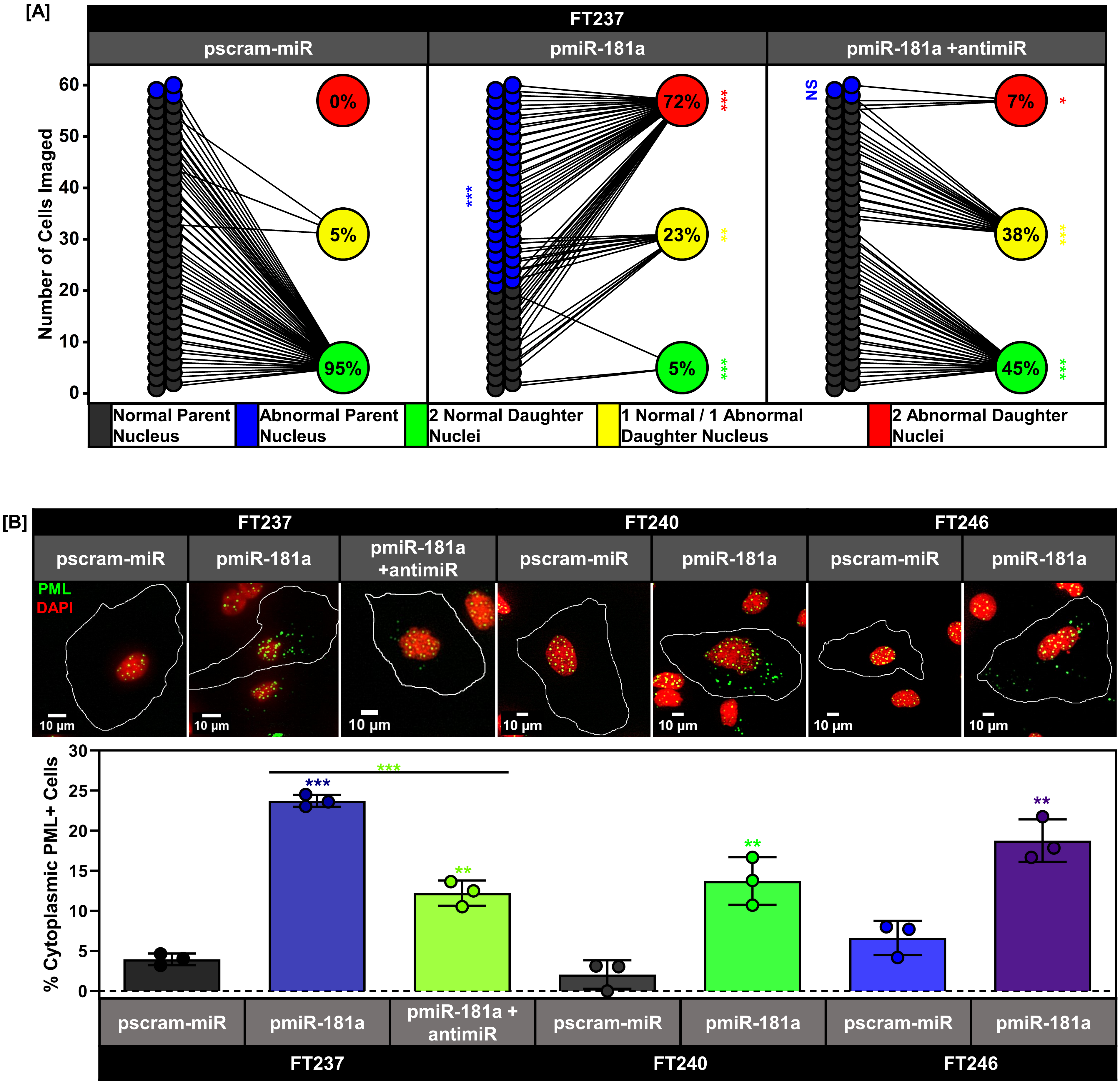

Supplementary Figure 3: miR-181a overexpression causes nuclear membrane rupture in FT237, FT240, and FT246 cells.
A) Plot showing nuclear shape status before and after cell division for FT237 pscram-miR, pmiR-181a, and pmiR-181a + antimiR cells with color key below. Chi-square analysis was used for statistical comparison between groups. B) Immunofluorescence micrographs of representative PML staining for the FT237 pscram-miR, pmiR-181a, pmiR-181a +antimiR, FT240 and FT246 pscram-miR and pmiR-181a cells. PML bodies are stained green, DAPI stained nuclei are colored red, and the outline of the cell is depicted in white. Quantification of the % cytoplasmic PML+ cells for each cell line is below. All data are representative of N = 3 independent experiments unless otherwise stated. The measure of center for the error bars is given as the mean value unless otherwise stated. The statistical test used for data analysis is the two-sided Student's t-test unless otherwise stated. Error bars indicate ±standard deviation unless otherwise stated. * = $p<0.05$, ** = $p<0.005$, *** = $p<0.0005$.

Supplementary Figure 4

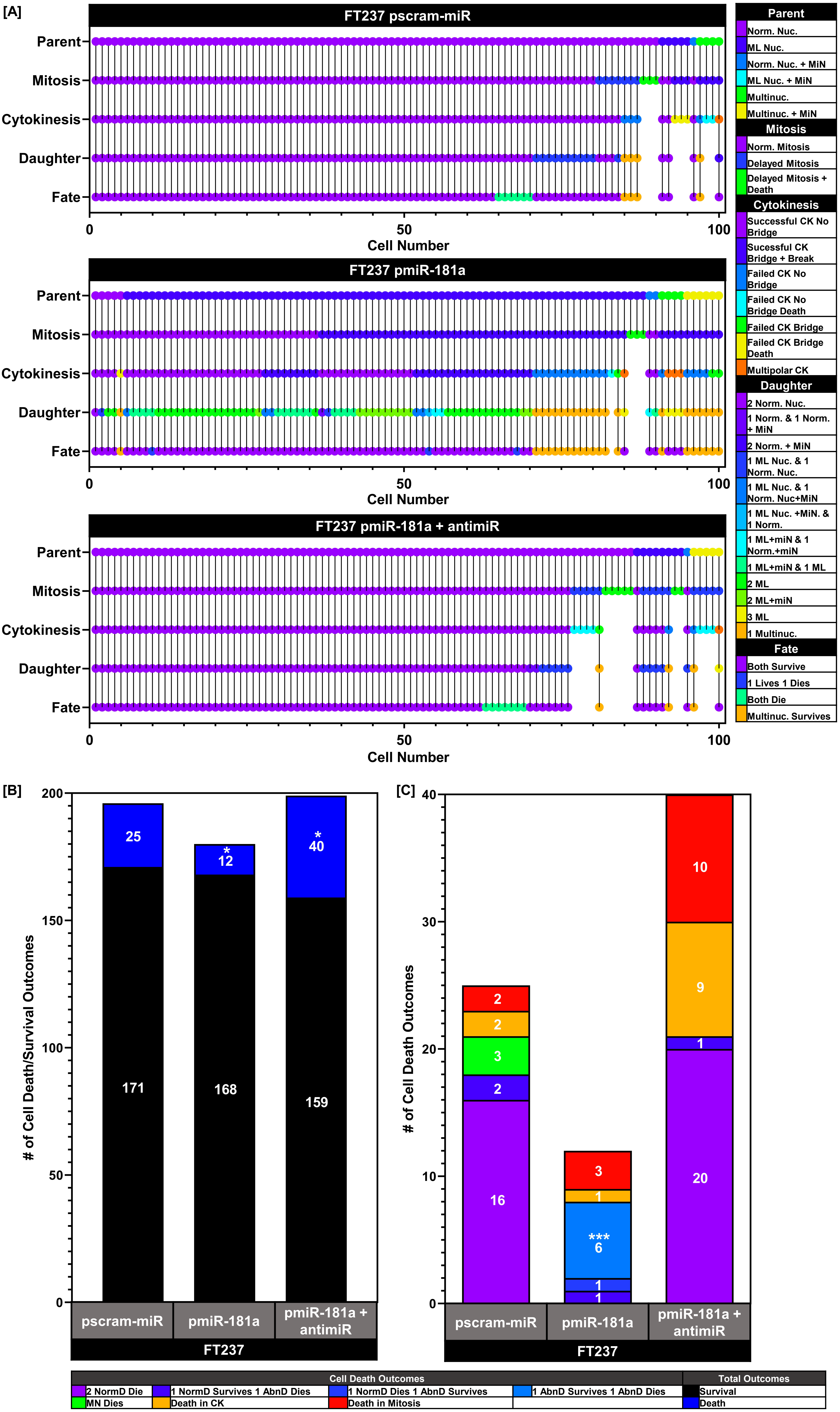

Supplementary Figure 4: miR-181a drives cytokinetic and mitotic defects without increasing death in FT237 cells

A) Graphs of detailed cell fate outcomes for FT237 pscram-miR, pmiR-181a, and pmiR-181a + antimiR cells. Each vertical set of connected dots represents a single cell undergoing division with the color of the dot representing a particular outcome at the corresponding stage of cell division. Color key with the various stages and outcomes is displayed on the right. B) Graph showing the number of cell death and cell survival outcomes for FT237 pscram-miR, pmiR-181a, and pmiR-181a + antimiR cells. C) Graph showing the number of cell death subtype outcomes for FT237 pscram-miR, pmiR-181a, and pmiR-181a + antimiR cells. Color keys for B) and C) are below. All data are representative of N = 3 independent experiments unless otherwise stated. The measure of center for the error bars is given as the mean value unless otherwise stated. Chi-square analysis was used for statistical comparison in 4B and 4C. The statistical test used for data analysis is the two-sided Student's t-test unless otherwise stated. Error bars indicate ±standard deviation unless otherwise stated. * = $p<0.05$, ** = $p<0.005$, *** = $p<0.0005$.

Supplementary Figure 5

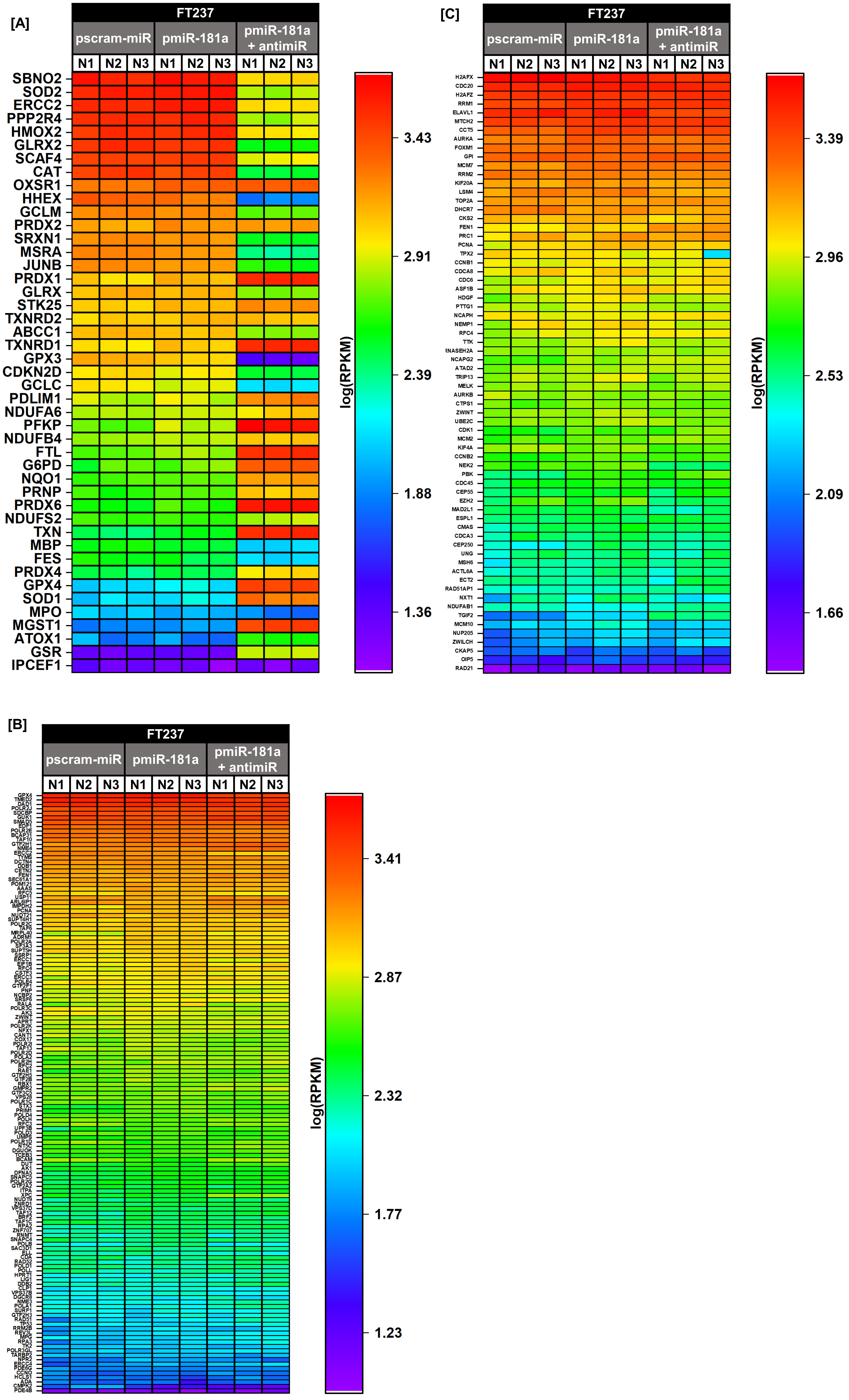

Supplementary Figure 5: GSEA of FT237 pmiR-181a cells shows enrichment of DNA damage and GI signatures
A-C) Full heatmaps of mRNA expression values from the DNA damage and GI GSEA analysis of FT237 microarray data. All data are representative of N = 3 independent experiments unless otherwise stated.

Supplementary Figure 6

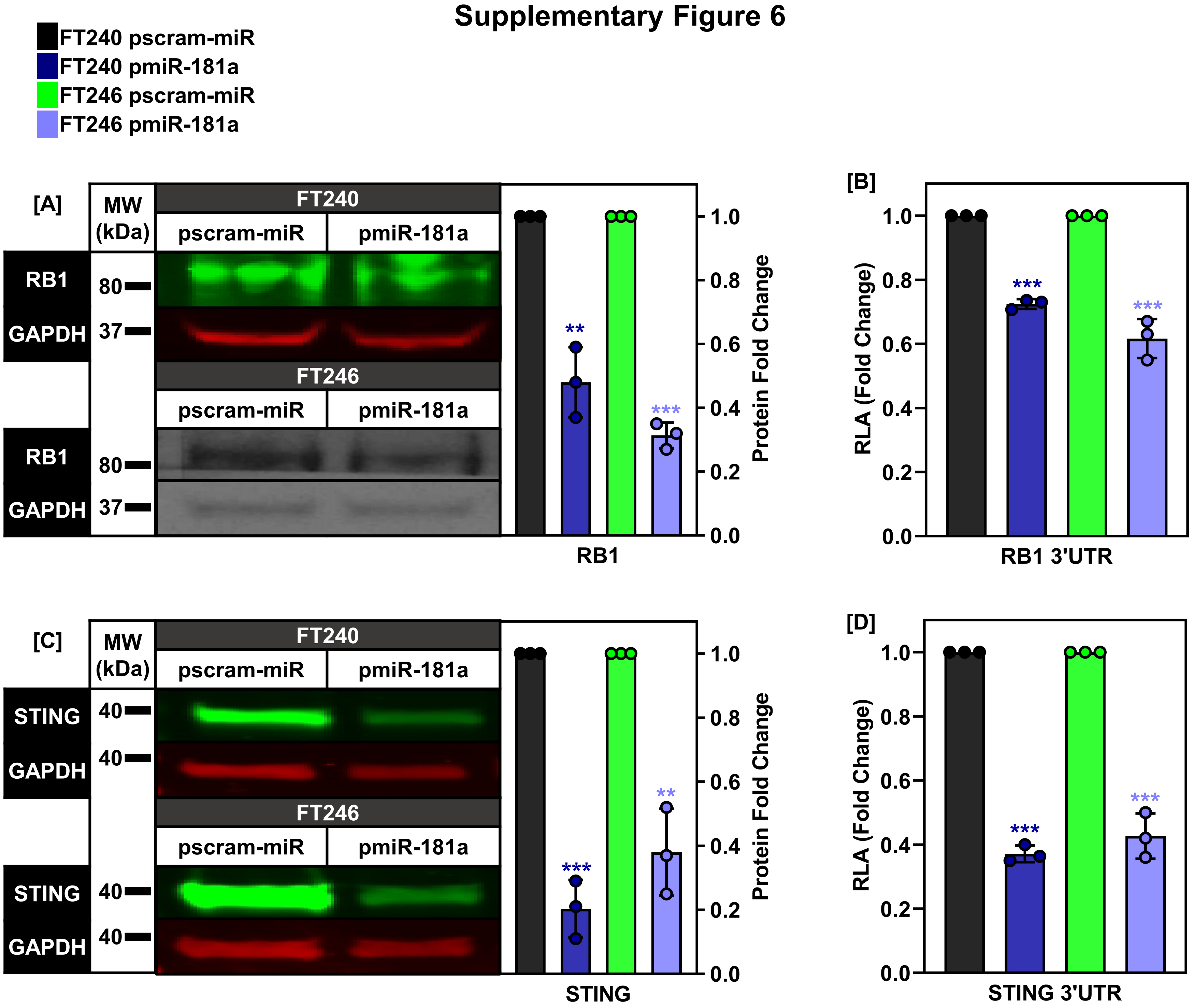

Supplementary Figure 6: miR-181a targets RB1 in FT240 and FT246 pmiR-181a cells

A) Representative western blots showing RB1 protein expression in the FT240 and FT246 pscram-miR and pmiR-181a cells. B) Quantification of RB1 protein expression in the FT240 and FT246 pscram-miR and pmiR-181a cells. C) Graph showing RB1 3'UTR relative luciferase activity for the FT240 and FT246 pscram-miR and pmiR-181a cells. D) Representative western blots showing STING protein expression in the FT240 and FT246 pscram-miR and pmiR-181a cells. E) Quantification of STING protein expression in the FT240 and FT246 pscram-miR and pmiR-181a cells. F) Graph showing STING 3'UTR relative luciferase activity for the FT240 and FT246 pscram-miR and pmiR-181a cells. All data are representative of N = 3 independent experiments unless otherwise stated. The measure of center for the error bars is given as the mean value unless otherwise stated. The statistical test used for data analysis is the two-sided Student's t-test unless otherwise stated. Error bars indicate ±standard deviation unless otherwise stated. * = $p<0.05$, ** = $p<0.005$, *** = $p<0.0005$. Full western blots shown in Supplemental Figure 12.

Supplementary Figure 7

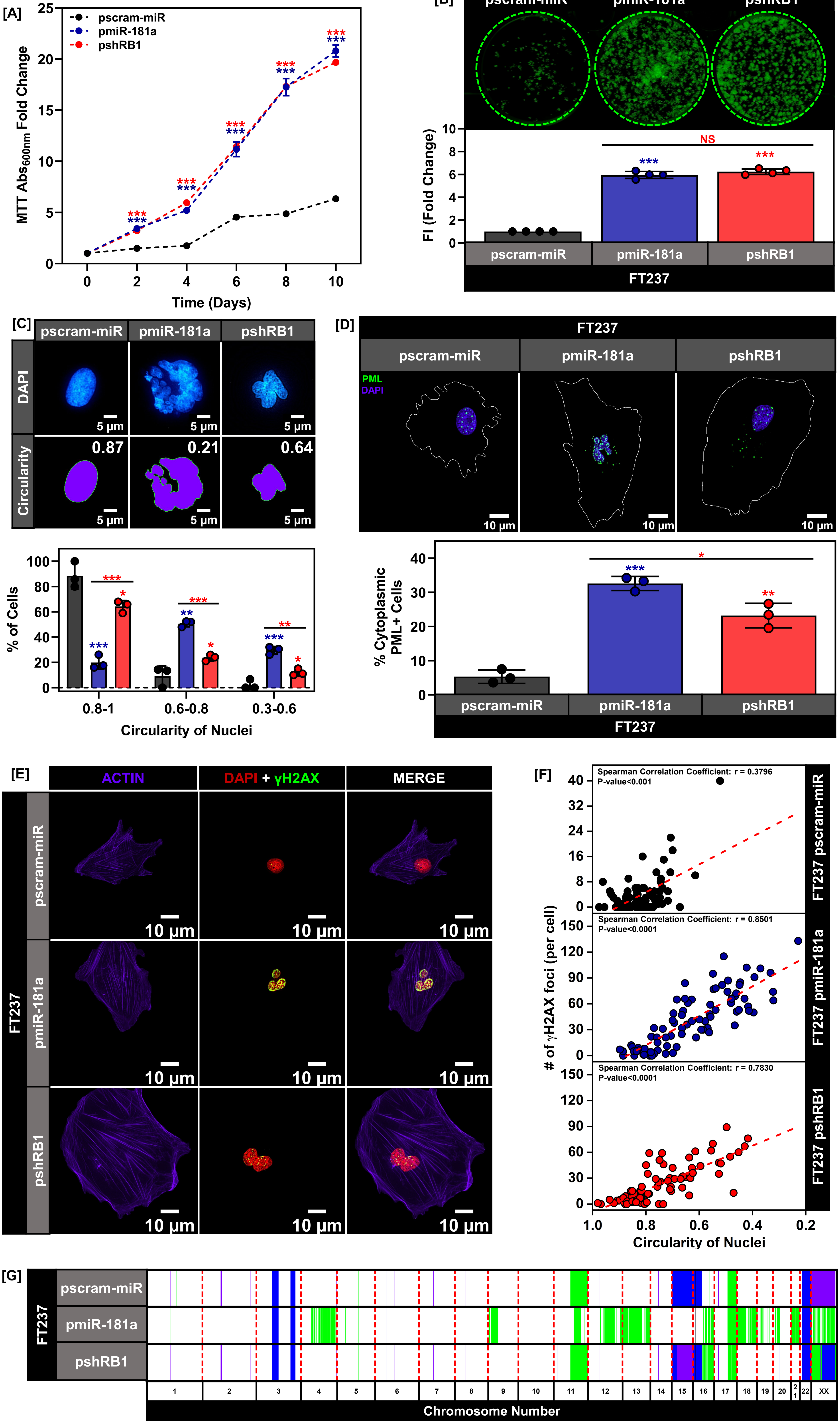

Supplementary Figure 7: Knockdown of RB1 in FTSECs phenocopies miR-181a mediated transformation

A) Graph showing increases in cell viability over a 10-day period for the FT237 pscram-miR, pmiR-181a, and pshRB1 cells. Significance values are color coded to match the pmiR-181a or pshRB1 cell line. B) Colony formation assay showing survival and colony formation for the FT237 pscram-miR, pmiR-181a, and pshRB1 cells with quantification below. Colonies were stained with CellTag 700 at 10 days. Dashed green lines denote the culture plate well boundaries. N = 4 for all cell lines. C) Immunofluorescence micrographs of representative DAPI stained nuclei (top), matched circularity masks with circularity value displayed in upper right hand corner (middle), and graph showing the circularity distribution (bottom) from FT237 pscram-miR, FT237 pmiR-181a, and FT237 pshRB1. D) Immunofluorescence micrographs of representative PML staining for the FT237 pscram-miR, pmiR-181a, and pshRB1 cells. PML bodies are stained green, DAPI stained nuclei are colored purple, and the outline of the cell is depicted in white. Quantification of the % cytoplasmic PML+ cells for each cell line is below. E) Representative immunofluorescence micrographs of γH2AX staining in FT237 pscram-miR, pmiR-181a, and pshRB1 cells. Purple indicates staining of actin with Actin-Red, red indicates staining of nuclei with DAPI, green indicates staining of γH2AX foci. F) Plots showing inverse correlation between the number of γH2AX foci for a cell nucleus and the corresponding circularity of the same cell nucleus for FT237 pscram-miR, pmiR-181a, and pshRB1 cells. Spearman's rank order correlation analysis was used for statistical analysis. Spearman correlation coefficients and p-values are displayed for each correlation plot. G) Genomap of copy number variants detected by SNP array in FT237 pscram-miR, pmiR-181a, and pshRB1 cells. All data are representative of N = 3 independent experiments unless otherwise stated. The measure of center for the error bars is given as the mean value unless otherwise stated. The statistical test used for data analysis is the two-sided Student's t-test unless otherwise stated. Error bars indicate ±standard deviation unless otherwise stated. * = $p<0.05$, ** = $p<0.005$, *** = $p<0.0005$.

Supplementary Figure 8

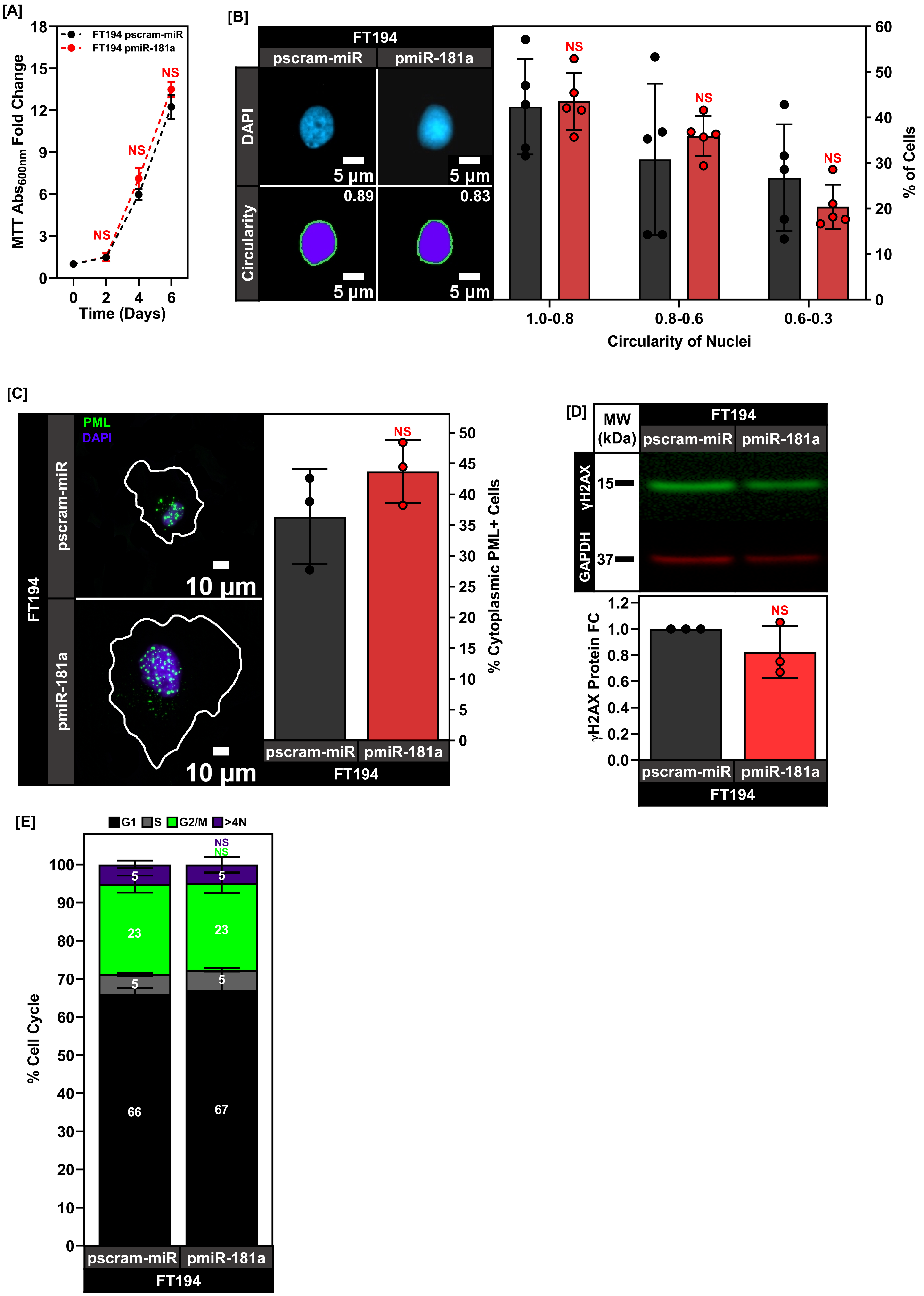

Supplementary Figure 8: SV40 large T transformed FTSECs do not display transformation phenotypes in response to miR-181a overexpression

A) Graph of cell viability growth curves for FT194 pscram-miR and pmiR-181a cells. B) Immunofluorescence micrographs of representative DAPI stained nuclei (top), matched circularity masks with circularity value displayed in upper right hand corner (bottom), and graph showing the circularity distribution (right) from FT194 pscram-miR and pmiR-181a cells. C) Immunofluorescence micrographs of representative PML staining for the FT194 pscram-miR and pmiR-181a cells. PML bodies are stained green, DAPI stained nuclei are colored purple, and the outline of the cell is depicted in white. Quantification of the % cytoplasmic PML+ cells for each cell line is below. D) Representative western blot of γH2AX protein expression in FT194 pscram-miR and pmiR-181a cells with quantification below. E) Graph showing quantification of cell cycle sub-populations for FT194 pscram-miR and pmiR-181a cells. All data are representative of N = 3 independent experiments unless otherwise stated. The measure of center for the error bars is given as the mean value unless otherwise stated. The statistical test used for data analysis is the two-sided Student's t-test unless otherwise stated. Error bars indicate ±standard deviation unless otherwise stated. * = $p<0.05$, ** = $p<0.005$, *** = $p<0.0005$. Full western blots shown in Supplemental Figure 12.

Supplementary Figure 9

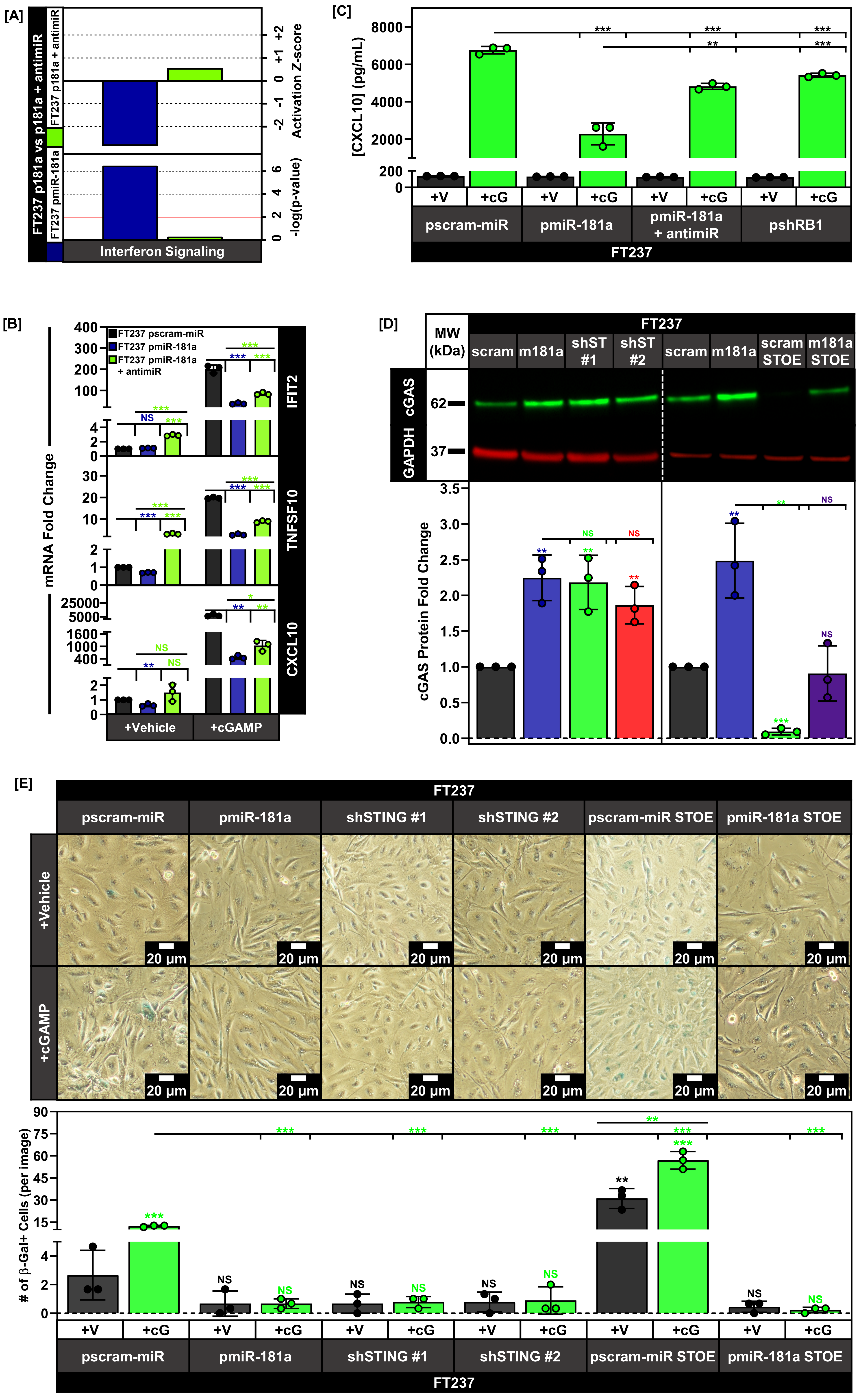

Supplementary Figure 9: miR-181a allows FTSECs to bypass GI-triggered innate immune activation by targeting the cytoplasmic DNA sensor STING

A) Graph comparing IPA Interferon Signaling in the FT237 pmiR-181a vs pmiR-181a & antimiR cells. (Bottom) graph of IPA Interferon Signaling –log(p-values) for the FT237 pmiR-181a and pmiR-181a + antimiR cells. (Top) graph of IPA Interferon Signaling Activation Z-scores for the FT237 pmiR-181a and pmiR-181a + antimiR cells. B) Graphs showing interferon inducible gene expression in FT237 pscram-miR, pmiR-181a, and pmiR-181a +antimiR cells in response to treatment with either lipofectamine vehicle or cGAMP. C) Representative western blots of cGAS expression in FT237 pscram-miR, pmiR-181a, shSTING #1, shSTING #2 pscram-miR STOE, and pmiR-181a STOE cells with quantification below. D) Bar graph showing secreted levels of CXCL10 for FT237 pscram-miR, pmiR-181a, pmiR-181a + antimiR, and pshRB1 cells 24 hours after treatment with either lipofectamine vehicle or lipofectamine + 10 ng/μL cGAMP. E) Representative micrographs of β-galactosidase staining in FT237 pscram-miR, pmiR-181a, shSTING #1, shSTING #2 pscram-miR STOE, and pmiR-181a STOE cells with quantification below. All data are representative of N = 3 independent experiments unless otherwise stated. The measure of center for the error bars is given as the mean value unless otherwise stated. The statistical test used for data analysis is the two-sided Student's t-test unless otherwise stated. Error bars indicate ±standard deviation unless otherwise stated. * = $p<0.05$, ** = $p<0.005$, *** = $p<0.0005$. Full western blots shown in Supplemental Figure 12.

Supplementary Figure 10

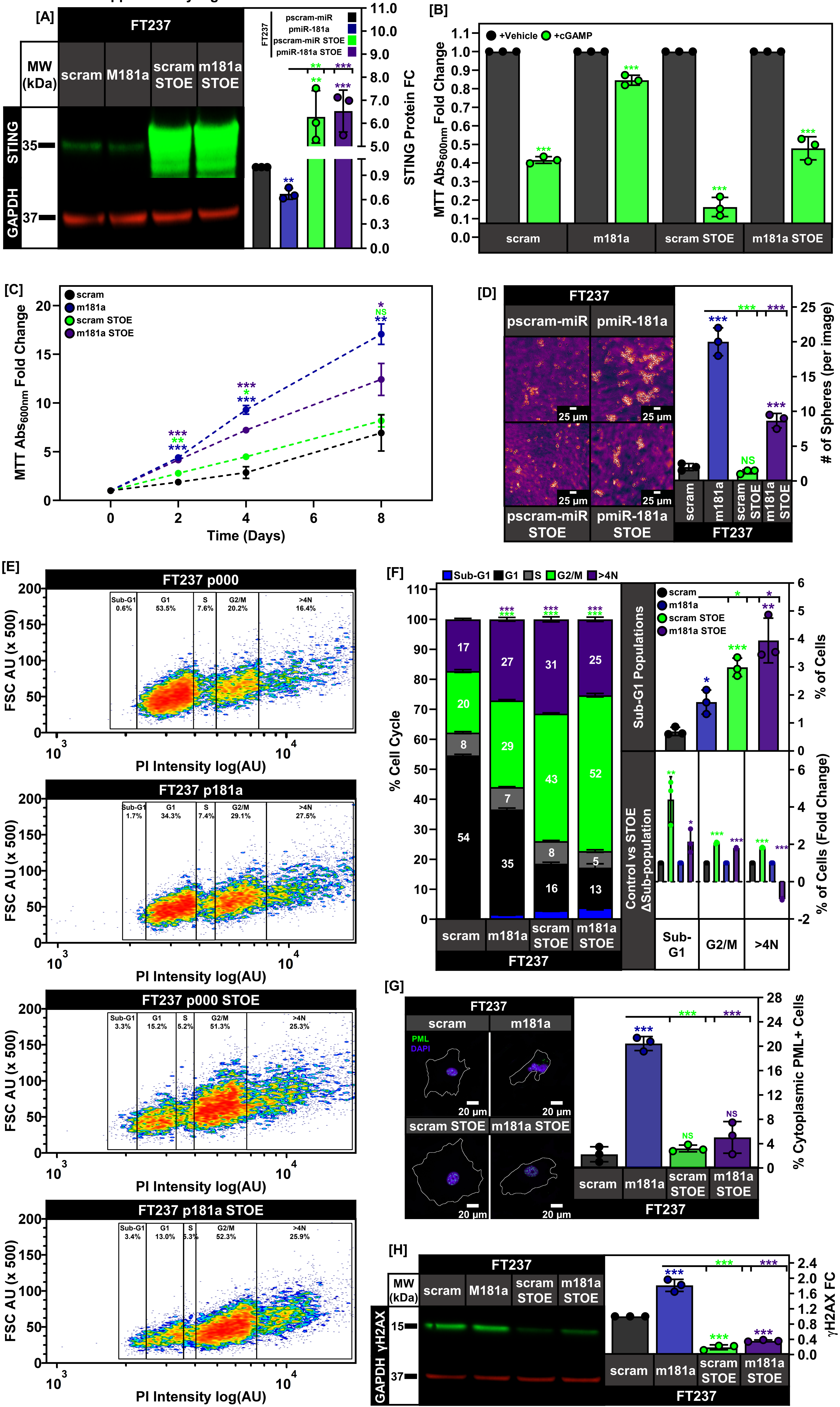

Supplementary Figure 10: Overexpression of STING inhibits miR-181a driven contributors to genomic instability and rescues activation of GI-induced interferon induced cell death

A) Representative western blot of STING protein expression in FT237 pscram-miR, pmiR-181a, pscram-miR STOE, and pmiR-181a STOE cells with quantification on the right. B) Graph of cell viability for FT237 pscram-miR, pmiR-181a, pscram-miR STOE, and pmiR-181a STOE cells 24 hours after treatment with either lipofectamine vehicle or lipofectamine + 10 ug of cGAMP. C) Graph of cell viability growth curves for FT237 pscram-miR, pmiR-181a, pscram-miR STOE, and pmiR-181a STOE cells. D) Micrographs showing anchorage independent growth of FT237 pscram-miR, pmiR-181a, pscram-miR STOE, and pmiR-181a STOE cells with quantification on the right. E) Representative graphs of cell cycle profiles for FT237 pscram-miR, pmiR-181a, pscram-miR STOE, and pmiR-181a STOE cells. F) Graphs depicting quantification of cell cycle subpopulations (left), magnified view of sub-G1 populations (top right), and change in subpopulation amount between control and STOE cells (bottom right) for FT237 pscram-miR, pmiR-181a, pscram-miR STOE, and pmiR-181a STOE cells. G) Immunofluorescence micrographs of representative PML staining for the FT237 pscram-miR, pmiR-181a, pscram-miR STOE, and pmiR-181a STOE cells. PML bodies are stained green, DAPI stained nuclei are colored purple, and the outline of the cell is depicted in white. Quantification of the % cytoplasmic PML+ cells for each cell line is located on the right. H) Representative western blot of γH2AX protein levels in the FT237 pscram-miR, pmiR-181a, pscram-miR STOE, and pmiR-181a STOE cells with quantification on the right. All data are representative of N = 3 independent experiments unless otherwise stated. The measure of center for the error bars is given as the mean value unless otherwise stated. The statistical test used for data analysis is the two-sided Student's t-test unless otherwise stated. Error bars indicate ±standard deviation unless otherwise stated. * = $p<0.05$, ** = $p<0.005$, *** = $p<0.0005$. Full western blots shown in Supplemental Figure 12.

Supplementary Figure 11

Figure 6B

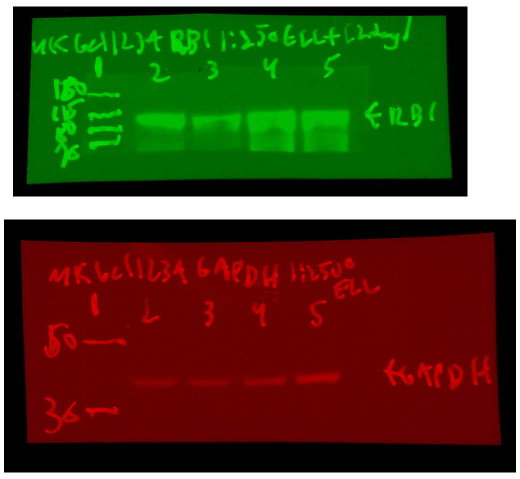

Images of film pseudocolored either green or red from original grayscaled image

Lane	Sample ID
1	Ladder
2	FT237 pscram-miR
3	FT237 pmiR-181a
4	FT237 pmiR-181a + antimiR
5	FT237 pmiR-181a + pMYC

Figure 7A

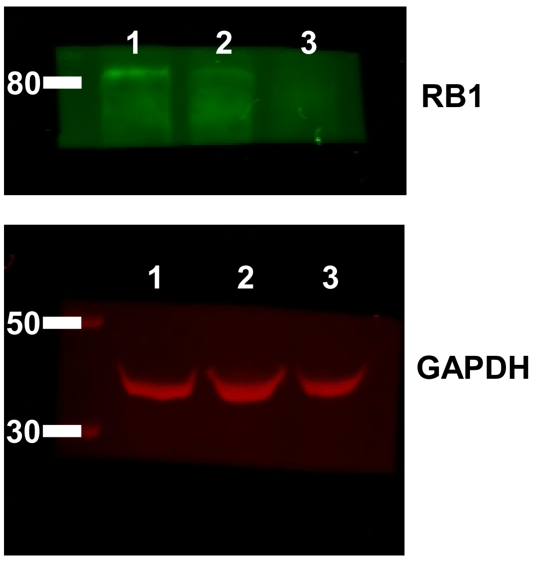

Lane	Sample ID
1	FT237 pscram-miR
2	FT237 pmiR-181a
3	FT237 pshRB1

Figure 8B

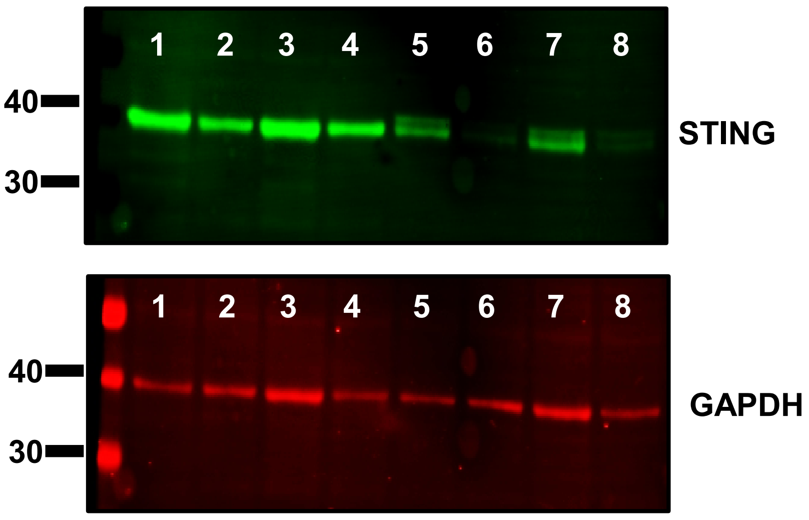

Lane	Sample ID
1	FT237 pscram-miR (+cGAMP)
2	FT237 pmiR-181a (+cGAMP)
3	FT237 pmiR-181a + antimiR (+cGAMP)
4	FT237 pshRB1 (+cGAMP)
5	FT237 pscram-miR (+vehicle)
6	FT237 pmiR-181a (+vehicle)
7	FT237 pmiR-181a + antimiR (+vehicle)
8	FT237 pshRB1 (+vehicle)

STING 1° Ab is Rabbit. GAPDH 1° Ab is mouse. LICOR Anti-Rabbit IRDye 800 2° Ab used to detect STING. LICOR Anti-Mouse IRDye 680 2° Ab used to detect GAPDH.

Figure 9A

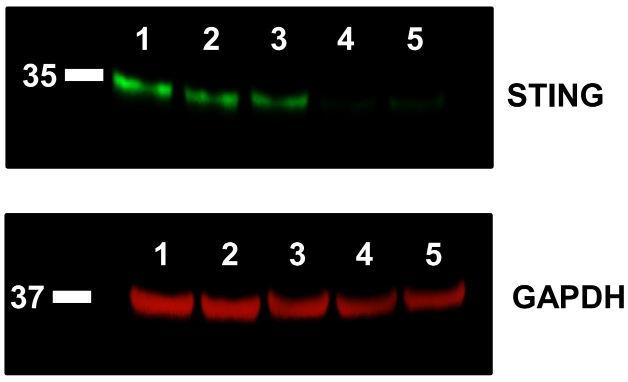

Lane	Sample ID
1	FT237 pscram-miR
2	FT237 pmiR-181a
3	FT237 shSTING #1
4	FT237 shSTING #2
5	FT237 shSTING #3

STING 1° Ab is Rabbit. GAPDH 1° Ab is mouse. LICOR Anti-Rabbit IRDye 800 2° Ab used to detect STING. LICOR Anti-Mouse IRDye 680 2° Ab used to detect GAPDH.

Figure 9I

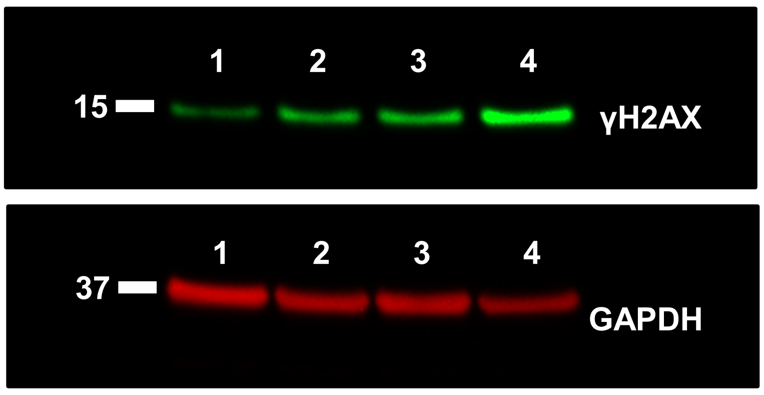

Lane	Sample ID
1	FT237 pscram-miR
2	FT237 pmiR-181a
3	FT237 shSTING #1
4	FT237 shSTING #2

Supplementary Figure 11
Full western blot images for Figures 6-9.

Supplementary Figure 12

Supplemental Figure 6A

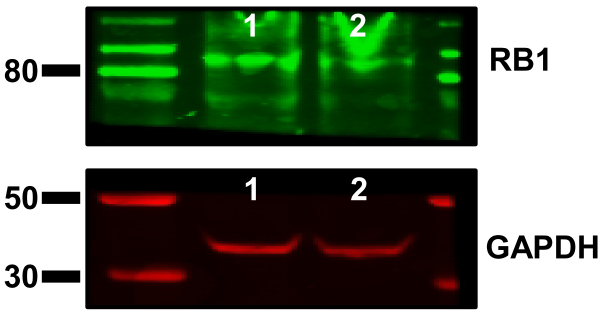

Lane	Sample ID
1	FT240 pscram-miR
2	FT240 pmiR-181a

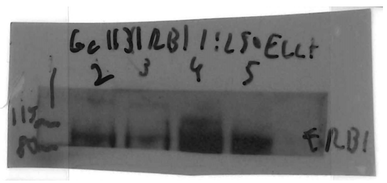

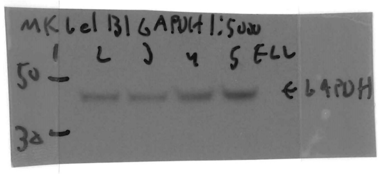

Lane	Sample ID
1	Ladder
2	FT246 pscram-miR N1
3	FT246 pmiR-181a N1
4	FT246 pscram-miR N2
5	FT246 pmiR-181a N2

Supplemental Figure 6C

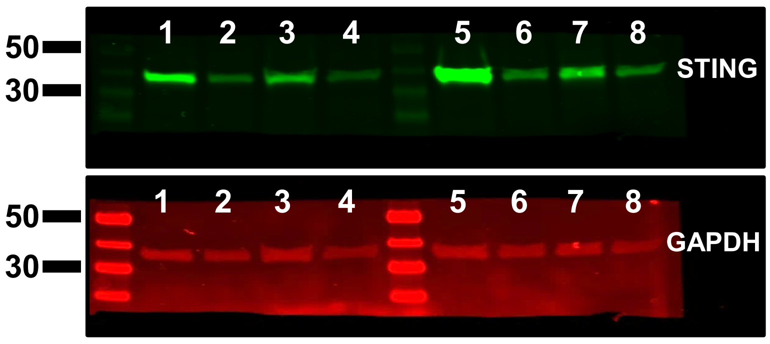

Lane	Sample ID
1	FT240 pscram-miR N1
2	FT240 pmiR-181a N1
3	FT240 pscram-miR N2
4	FT240 pmiR-181a N2
5	FT246 pscram-miR N1
6	FT246 pmiR-181a N1
7	FT246 pscram-miR N2
8	FT246 pmiR-181a N2

STING 1° Ab is Rabbit. GAPDH 1° Ab is mouse. LICOR Anti-Rabbit IRDye 800 2° Ab used to detect STING. LICOR Anti-Mouse IRDye 680 2° Ab used to detect GAPDH.

Supplemental Figure 8D

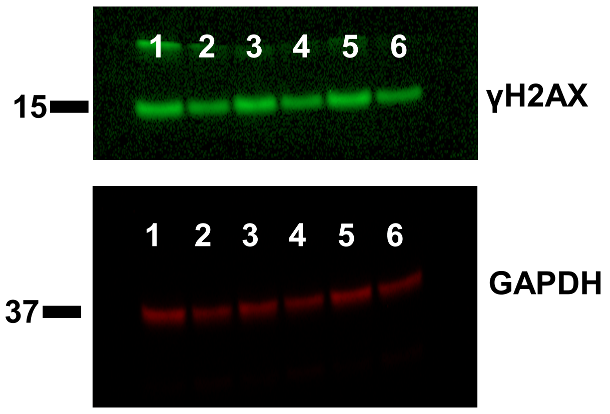

Lane	Sample ID
1	FT194 pscram-miR N1
2	FT194 pmiR-181a N1
3	FT194 pscram-miR N2
4	FT194 pmiR-181a N2
5	FT194 pscram-miR N3
6	FT194 pmiR-181a N3

Supplemental Figure 10A

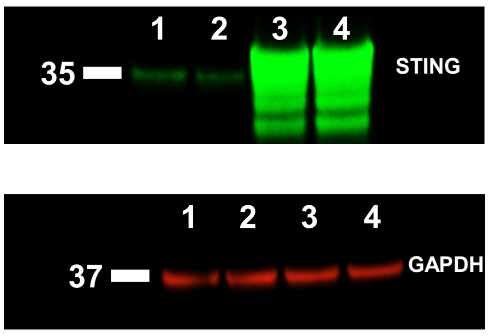

Lane	Sample ID
1	FT237 pscram-miR
2	FT237 pmiR-181a
3	FT237 pscram-miR STING OE
4	FT237 pmiR-181a STING OE

STING 1° Ab is Rabbit. GAPDH 1° Ab is mouse. LICOR Anti-Rabbit IRDye 800 2° Ab used to detect STING. LICOR Anti-Mouse IRDye 680 2° Ab used to detect GAPDH.

Supplemental Figure 9D

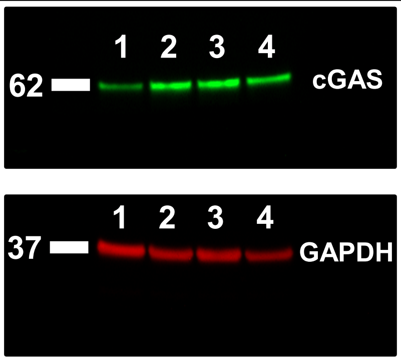

Lane	Sample ID
1	FT237 pscram-miR
2	FT237 pmiR-181a
3	FT237 shSTING #1
4	FT237 shSTING #2

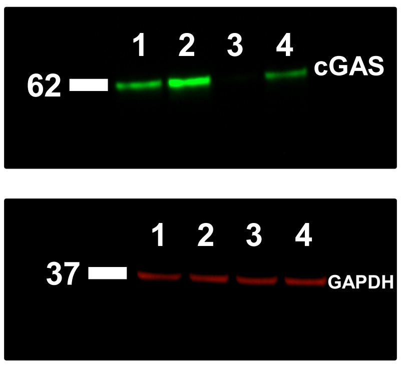

Lane	Sample ID
1	FT237 pscram-miR
2	FT237 pmiR-181a
3	FT237 pscram-miR STING OE
4	FT237 pmiR-181a STING OE

Supplemental Figure 10H

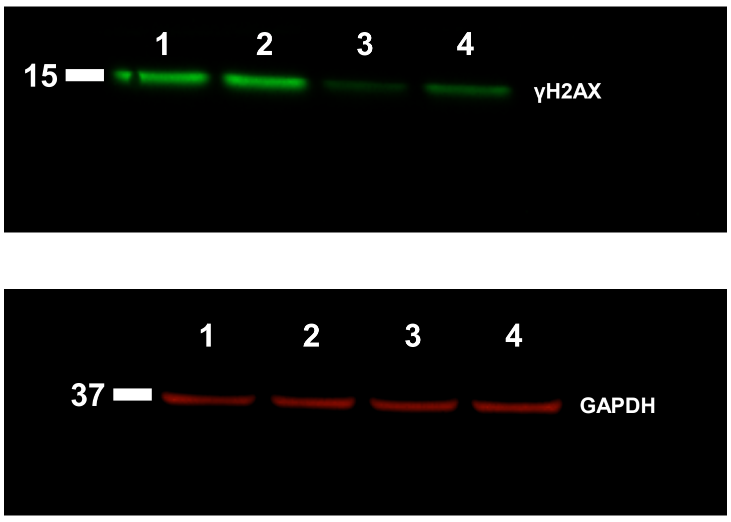

Lane	Sample ID
1	FT237 pscram-miR
2	FT237 pmiR-181a
3	FT237 pscram-miR STING OE
4	FT237 pmiR-181a STING OE

Supplementary Figure 12
Full western blot images for Supplemental Figures 6, 8, 9, and 10

Supplemental Table 1: List of Primers Used	
**18S RP**	GGAAAGCAGACATTGACCTCAC
**18S LP**	CCATCCTTTACATCCTTCTGTCTGT
**RB1 RP**	GTTGGTCCTTCTCGGTCCTT
**RB1 LP**	CAAAGCAGAAGGCAACTTGA
**STING FP**	CACTTGGATGCTTGCCCTC
**STING RP**	GCCACGTTGAAATTCCCTTTTT
**IFIT2 FP**	AAGCACCTCAAAGGGCAAAAC
**IFIT2 RP**	TCGGCCCATGTGATAGTAGAC
**CXCL10 FP**	GTGGCATTCAAGGAGTACCTC
**CXCL10 RP**	TGATGGCCTTCGATTCTGGATT
**TNFSF10 FP**	TGCGTGCTGATCGTGATCTTC
**TNFSF10 RP**	GCTCGTTGGTAAAGTACACGTA

Supplemental Table 2: List of Antibodies Used			
**Primary Antibody**	Company	Catalog #	Dilution
**RB1**	Cell Signaling Technology	9313	1:500
**GAPDH**	Santa Cruz	sc-365062	1:2500
**STING**	Cell Signaling Technology	13647S	1:500
**IFIT2**	Santa Cruz	Sc-390724	1:250
**TNFSF10**	Cell Signaling Technology	3219S	1:500
**CXCL10**	Abcam	Ab8098	1:500
**Secondary Antibody**	**Company**	**Catalog #**	**Dilution**
**IRDye® 800CW Goat anti-Rabbit IgG**	LICOR	925-32211	1:2500
**IRDye® 680LT Goat anti-Rabbit IgG**	LICOR	925-68021	1:2500
**IRDye® 800CW Goat anti-Mouse IgG**	LICOR	925-32210	1:2500
**IRDye® 680LT Goat anti-Mouse IgG**	LICOR	925-68020	1:2500

Supplemental Table 3: Figure 1 Statistical Tests

Figure Panel	Groups Compared	Statistical Test Used	P value	P value summary	Significantly different (P < 0.05)?	One- or two-tailed P value?	t, df	Difference of Means 95% Confidence Interval
1B	FT237 pscram-miR vs pmiR-181a	Unpaired t test	0.0006	***	Yes	Two-tailed	t=9.950, df=4	**27.76 to 49.24**
1B	FT240 pscram-miR vs pmiR-181a	Unpaired t test	0.0027	**	Yes	Two-tailed	t=6.601, df=4	**12.65 to 31.02**
1B	FT246 pscram-miR vs pmiR-181a	Unpaired t test	0.0012	**	Yes	Two-tailed	t=8.299, df=4	**19.72 to 39.55**
1C	FT237 pscram-miR vs pmiR-181a D2	Unpaired t test	0.025	*	Yes	Two-tailed	t=3.496, df=4	**0.04744 to 0.4136**
1C	FT237 pscram-miR vs pmiR-181a D4	Unpaired t test	0.0002	***	Yes	Two-tailed	t=12.97, df=4	1.328 to 2.051
1C	FT237 pscram-miR vs pmiR-181a D6	Unpaired t test	<0.0001	****	Yes	Two-tailed	t=17.48, df=4	3.990 to 5.496
1C	FT237 pscram-miR vs pmiR-181a D8	Unpaired t test	<0.0001	****	Yes	Two-tailed	t=16.55, df=4	6.081 to 8.532
1C	FT237 pscram-miR vs pmiR-181a D10	Unpaired t test	0.0003	***	Yes	Two-tailed	t=12.30, df=4	7.804 to 12.35
1C	FT240 pscram-miR vs pmiR-181a D2	Unpaired t test	<0.0001	****	Yes	Two-tailed	t=20.96, df=4	1.323 to 1.727
1C	FT240 pscram-miR vs pmiR-181a D4	Unpaired t test	<0.0001	****	Yes	Two-tailed	t=24.45, df=4	6.341 to 7.965
1C	FT240 pscram-miR vs pmiR-181a D6	Unpaired t test	<0.0001	****	Yes	Two-tailed	t=27.32, df=4	8.236 to 10.10
1C	FT240 pscram-miR vs pmiR-181a D8	Unpaired t test	<0.0001	****	Yes	Two-tailed	t=22.39, df=4	13.96 to 17.91
1C	FT240 pscram-miR vs pmiR-181a D10	Unpaired t test	<0.0001	****	Yes	Two-tailed	t=22.31, df=4	14.49 to 18.61
1C	FT246 pscram-miR vs pmiR-181a D2	Unpaired t test	<0.0001	****	Yes	Two-tailed	t=32.06, df=4	1.565 to 1.862
1C	FT246 pscram-miR vs pmiR-181a D4	Unpaired t test	<0.0001	****	Yes	Two-tailed	t=17.72, df=4	5.332 to 7.313
1C	FT246 pscram-miR vs pmiR-181a D6	Unpaired t test	0.0002	***	Yes	Two-tailed	t=12.66, df=4	4.221 to 6.592
1C	FT246 pscram-miR vs pmiR-181a D8	Unpaired t test	0.0005	***	Yes	Two-tailed	t=10.33, df=4	6.215 to 10.78
1C	FT246 pscram-miR vs pmiR-181a D10	Unpaired t test	0.0002	***	Yes	Two-tailed	t=12.53, df=4	7.052 to 11.07
1D	FT237 pscram-miR vs pmiR-181a	Unpaired t test	0.0002	***	Yes	Two-tailed	t=12.40, df=4	4.494 to 7.086
1D	FT240 pscram-miR vs pmiR-181a	Unpaired t test	<0.0001	****	Yes	Two-tailed	t=17.73, df=4	1.521 to 2.086
1D	FT246 pscram-miR vs pmiR-181a	Unpaired t test	0.0055	**	Yes	Two-tailed	t=5.448, df=4	5.476 to 16.86
1E	FT237 pscram-miR vs pmiR-181a	Unpaired t test	<0.0001	****	Yes	Two-tailed	t=9.858, df=8	14.25 to 22.95
1E	FT240 pscram-miR vs pmiR-181a	Unpaired t test	<0.0001	****	Yes	Two-tailed	t=18.23, df=8	32.01 to 41.29
1E	FT246 pscram-miR vs pmiR-181a	Unpaired t test	<0.0001	****	Yes	Two-tailed	t=8.285, df=8	16.31 to 28.89
1F	FT237 pscram-miR vs pmiR-181a G2/M	Unpaired t test	<0.0001	****	Yes	Two-tailed	t=16.82, df=4	12.87 to 17.96
1F	FT240 pscram-miR vs pmiR-181a G2/M	Unpaired t test	0.0004	***	Yes	Two-tailed	t=10.63, df=4	8.286 to 14.14
1F	FT246 pscram-miR vs pmiR-181a G2/M	Unpaired t test	0.0412	*	Yes	Two-tailed	t=2.967, df=4	0.3338 to 10.03
1F	FT237 pscram-miR vs pmiR-181a >4N	Unpaired t test	0.0007	***	Yes	Two-tailed	t=9.353, df=4	4.217 to 7.778
1F	FT240 pscram-miR vs pmiR-181a >4N	Unpaired t test	0.0053	**	Yes	Two-tailed	t=5.498, df=4	1.928 to 5.863
1F	FT246 pscram-miR vs pmiR-181a >4N	Unpaired t test	0.001	**	Yes	Two-tailed	t=8.584, df=4	4.564 to 8.927

Supplemental Table 4: Figure 2 Statistical Tests

Figure Panel	Groups Compared	Statistical Test Used	P value	P value summary	Significantly different (P < 0.05)?	One- or two-tailed P value?			
**2B**	**FT237 pscram-miR vs pmiR-181a**	Fisher's exact test	0.0001	***	Yes	Two-sided			
**2B**	**FT237 pscram-miR vs antimiR**	Fisher's exact test	>0.9999	ns	No	Two-sided			

Figure Panel	Groups Compared	Statistical Test Used	P value	Exact or approximate P value?	P value summary	Significantly different (P < 0.05)?	One- or two-tailed P value?	Sum of ranks in column A,B	Mann-Whitney U
**2C**	**FT237 pscram-miR vs pmiR-181a WK2**	Mann Whitney U test	0.0971	Exact	ns	No	Two-tailed	83 , 127	28
**2C**	**FT237 pscram-miR vs antimiR WK2**	Mann Whitney U test	0.0016	Exact	**	Yes	Two-tailed	65.50 , 144.5	10.5
**2C**	**FT237 pscram-miR vs pmiR-181a WK4**	Mann Whitney U test	0.0002	Exact	***	Yes	Two-tailed	60.50 , 149.5	5.5
**2C**	**FT237 pscram-miR vs antimiR WK4**	Mann Whitney U test	0.0015	Exact	**	Yes	Two-tailed	66 , 144	11
**2C**	**FT237 pscram-miR vs pmiR-181a WK6**	Mann Whitney U test	0.0002	Exact	***	Yes	Two-tailed	61 , 149	6
**2C**	**FT237 pscram-miR vs antimiR WK6**	Mann Whitney U test	0.0035	Exact	**	Yes	Two-tailed	69.50 , 140.5	14.5
**2C**	**FT237 pscram-miR vs pmiR-181a WK8**	Mann Whitney U test	0.0001	Exact	***	Yes	Two-tailed	60 , 150	5
**2C**	**FT237 pscram-miR vs antimiR WK8**	Mann Whitney U test	0.0007	Exact	***	Yes	Two-tailed	65 , 145	10
**2C**	**FT237 pscram-miR vs pmiR-181a WK10**	Mann Whitney U test	0.0001	Exact	***	Yes	Two-tailed	60 , 150	5
**2C**	**FT237 pscram-miR vs antimiR WK10**	Mann Whitney U test	0.0108	Exact	*	Yes	Two-tailed	75 , 135	20
**2C**	**FT237 pscram-miR vs pmiR-181a WK12**	Mann Whitney U test	0.0001	Exact	***	Yes	Two-tailed	60 , 150	5
**2C**	**FT237 pscram-miR vs antimiR WK12**	Mann Whitney U test	0.0108	Exact	*	Yes	Two-tailed	75 , 135	20
**2C**	**FT237 pscram-miR vs pmiR-181a WK14**	Mann Whitney U test	0.0001	Exact	***	Yes	Two-tailed	60 , 150	5
**2C**	**FT237 pscram-miR vs antimiR WK14**	Mann Whitney U test	0.0108	Exact	*	Yes	Two-tailed	75 , 135	20
**2C**	**FT237 pscram-miR vs pmiR-181a WK16**	Mann Whitney U test	0.0001	Exact	***	Yes	Two-tailed	60 , 150	5
**2C**	**FT237 pscram-miR vs antimiR WK16**	Mann Whitney U test	0.0325	Exact	*	Yes	Two-tailed	80 , 130	25
**2C**	**FT237 pscram-miR vs pmiR-181a WK18**	Mann Whitney U test	0.0001	Exact	***	Yes	Two-tailed	60 , 150	5
**2C**	**FT237 pscram-miR vs antimiR WK18**	Mann Whitney U test	0.0867	Exact	ns	No	Two-tailed	85 , 125	30
**2C**	**FT237 pscram-miR vs pmiR-181a WK 20**	Mann Whitney U test	0.0001	Exact	***	Yes	Two-tailed	60 , 150	5
**2C**	**FT237 pscram-miR vs antimiR WK20**	Mann Whitney U test	0.0867	Exact	ns	No	Two-tailed	85 , 125	30
**2C**	**FT237 pscram-miR vs pmiR-181a WK22**	Mann Whitney U test	0.0001	Exact	***	Yes	Two-tailed	60 , 150	5
**2C**	**FT237 pscram-miR vs antimiR WK22**	Mann Whitney U test	0.2105	Exact	ns	No	Two-tailed	90 , 120	35
**2C**	**FT237 pscram-miR vs pmiR-181a WK25**	Mann Whitney U test	0.0001	Exact	***	Yes	Two-tailed	60 , 150	5
**2C**	**FT237 pscram-miR vs antimiR WK25**	Mann Whitney U test	0.2105	Exact	ns	No	Two-tailed	90 , 120	35
**2D**	**FT237 pscram-miR vs pmiR-181a**	Mann Whitney U test	0.0001	Exact	***	Yes	Two-tailed	60 , 150	5
**2D**	**FT237 pscram-miR vs antimiR**	Mann Whitney U test	>0.9999	Exact	ns	No	Two-tailed	100 , 110	45
**2D**	**FT237 pmiR-181a vs antimiR**	Mann Whitney U test	0.0001	Exact	***	Yes	Two-tailed	149.5 , 60.50	5.5

Figure Panel	Groups Compared	Statistical Test Used	Chi-square, df	z	P value	P value summary	One- or two-sided	Statistically significant (P < 0.05)?	
**2G**	**FT237 pscram-miR vs pmiR-181a**	Chi Square	3.692, 1	1.922	0.0547	ns	Two-sided	No	
**2G**	**FT237 pscram-miR vs pmiR-181a**	Chi Square	3.692, 1	1.922	0.0547	ns	Two-sided	No	

Figure Panel	Groups Compared	Statistical Test Used	P value	Exact or approximate P value?	P value summary	Significantly different (P < 0.05)?	One- or two-tailed P value?	Sum of ranks in column A,B	Mann-Whitney U
**2H**	**FT237 pscram-miR vs pmiR-181a**	Mann Whitney U test	0.2	Exact	ns	No	Two-tailed	56 , 80	20

Supplemental Table 5: Figure 3 Statistical Tests

Figure Panel	Groups Compared	Statistical Test Used	P value	P value summary	Significantly different (P < 0.05)?	One- or two-tailed P value?	t, df	Difference of Means 95% Confidence Interval
3A	FT237 pscram-miR vs pmiR-181a 1-0.8	Unpaired t test	0.003	**	Yes	Two-tailed	t=6.442, df=4	-54.94 to -21.84
3A	FT237 pscram-miR vs antimiR 1-0.8	Unpaired t test	0.9683	ns	No	Two-tailed	t=0.04234, df=4	-8.134 to 7.890
3A	FT237 pmiR-181a vs antimiR 1-0.8	Unpaired t test	0.0034	**	Yes	Two-tailed	t=6.220, df=4	21.19 to 55.35
3A	FT237 pscram-miR vs pmiR-181a 0.8-0.6	Unpaired t test	0.0081	**	Yes	Two-tailed	t=4.886, df=4	6.744 to 24.50
3A	FT237 pscram-miR vs antimiR 0.8-0.6	Unpaired t test	0.1913	ns	No	Two-tailed	t=1.571, df=4	-16.39 to 4.546
3A	FT237 pmiR-181a vs antimiR 0.8-0.6	Unpaired t test	0.0077	**	Yes	Two-tailed	t=4.966, df=4	-33.59 to -9.498
3A	FT237 pscram-miR vs pmiR-181a 0.6-0.3	Unpaired t test	0.0006	***	Yes	Two-tailed	t=9.957, df=4	15.04 to 26.68
3A	FT237 pscram-miR vs antimiR 0.6-0.3	Unpaired t test	0.0386	*	Yes	Two-tailed	t=3.034, df=4	0.4402 to 9.939
3A	FT237 pmiR-181a vs antimiR 0.6-0.3	Unpaired t test	0.0035	**	Yes	Two-tailed	t=6.164, df=4	-22.73 to -8.612
3C	FT237 pscram-miR vs pmiR-181a	Unpaired t test	<0.0001	****	Yes	Two-tailed	t=11.72, df=8	42.09 to 62.71
3C	FT237 pscram-miR vs antimiR	Unpaired t test	0.0005	***	Yes	Two-tailed	t=5.688, df=8	4.495 to 10.62
3C	FT237 pmiR-181a vs antimiR	Unpaired t test	<0.0001	****	Yes	Two-tailed	t=9.769, df=8	-55.42 to -34.26
3D	FT237 pscram-miR vs pmiR-181a	Unpaired t test	<0.0001	****	Yes	Two-tailed	t=6.129, df=146	1.630 to 3.181
3D	FT237 pscram-miR vs antimiR	Unpaired t test	0.0674	ns	No	Two-tailed	t=1.843, df=146	-0.007844 to 0.2241
3D	FT237 pmiR-181a vs antimiR	Unpaired t test	<0.0001	****	Yes	Two-tailed	t=5.802, df=146	-3.080 to -1.515
3E	FT237 pscram-miR vs pmiR-181a	Unpaired t test	<0.0001	****	Yes	Two-tailed	t=7.359, df=118	51.79 to 89.91
3E	FT237 pscram-miR vs antimiR	Unpaired t test	0.0181	*	Yes	Two-tailed	t=2.398, df=118	-21.52 to -2.051
3E	FT237 pmiR-181a vs antimiR	Unpaired t test	<0.0001	****	Yes	Two-tailed	t=8.422, df=118	-102.1 to -63.20
3F	FT237 pscram-miR vs pmiR-181a LC	Unpaired t test	<0.0001	****	Yes	Two-tailed	t=11.69, df=6	15.67 to 23.96
3F	FT237 pscram-miR vs antimiR LC	Unpaired t test	0.6394	ns	No	Two-tailed	t=0.4933, df=6	-2.242 to 3.375
3F	FT237 pmiR-181a vs antimiR LC	Unpaired t test	<0.0001	****	Yes	Two-tailed	t=11.34, df=6	-23.40 to -15.09
3F	FT237 pscram-miR vs pmiR-181a NB	Unpaired t test	<0.0001	****	Yes	Two-tailed	t=9.625, df=6	19.99 to 33.62
3F	FT237 pscram-miR vs antimiR NB	Unpaired t test	0.4575	ns	No	Two-tailed	t=0.7938, df=6	-1.833 to 3.593
3F	FT237 pmiR-181a vs antimiR NB	Unpaired t test	<0.0001	****	Yes	Two-tailed	t=9.521, df=6	-32.59 to -19.26
3F	FT237 pscram-miR vs pmiR-181a FC	Unpaired t test	0.0005	***	Yes	Two-tailed	t=6.695, df=6	9.604 to 20.67
3F	FT237 pscram-miR vs antimiR FC	Unpaired t test	0.889	ns	No	Two-tailed	t=0.1456, df=6	-1.123 to 0.9968
3F	FT237 pmiR-181a vs antimiR FC	Unpaired t test	0.0005	***	Yes	Two-tailed	t=6.737, df=6	-20.72 to -9.679
3F	FT237 pscram-miR vs pmiR-181a MC	Unpaired t test	<0.0001	****	Yes	Two-tailed	t=16.00, df=6	4.282 to 5.828
3F	FT237 pscram-miR vs antimiR MC	Unpaired t test	0.3559	ns	No	Two-tailed	t=1.000, df=6	-0.3617 to 0.8617
3F	FT237 pmiR-181a vs antimiR MC	Unpaired t test	<0.0001	****	Yes	Two-tailed	t=11.93, df=6	-5.791 to -3.819
**Figure Panel**	**Groups Compared**	**Statistical Test Used**	**Chi-square, df**	**z**	**P value**	**P value summary**	**One- or two-sided**	**Statistically significant (P < 0.05)?**
3G	FT237 pscram-miR vs pmiR-181a DP	Chi Square	7.254, 1	2.693	0.0071	**	Two-sided	Yes
3G	FT237 pscram-miR vs antimiR DP	Chi Square	0.000, 1	0	>0.9999	ns	Two-sided	No
3G	FT237 pscram-miR vs pmiR-181a P	Chi Square	116.2, 1	10.78	<0.0001	****	Two-sided	Yes
3G	FT237 pscram-miR vs antimiR P	Chi Square	2.696, 1	1.642	0.1006	ns	Two-sided	No
3G	FT237 pscram-miR vs pmiR-181a B	Chi Square	1.846, 1	1.359	0.1742	ns	Two-sided	No
3G	FT237 pscram-miR vs antimiR B	Chi Square	3.532, 1	1.879	0.0602	ns	Two-sided	No
3G	FT237 pscram-miR vs pmiR-181a G	Chi Square	1.005, 1	1.003	0.3161	ns	Two-sided	No
3G	FT237 pscram-miR vs antimiR G	Chi Square	NA	NA	NA	NA	NA	NA
3G	FT237 pscram-miR vs pmiR-181a Y	Chi Square	6.186, 1	2.487	0.0129	*	Two-sided	Yes
3G	FT237 pscram-miR vs antimiR Y	Chi Square	NA	NA	NA	NA	NA	NA
3G	FT237 pscram-miR vs pmiR-181a BO	Chi Square	87.77, 1	9.369	<0.0001	****	Two-sided	Yes
3G	FT237 pscram-miR vs antimiR BO	Chi Square	NA	NA	NA	NA	NA	NA
3G	FT237 pscram-miR vs pmiR-181a DO	Chi Square	13.21, 1	3.635	0.0003	***	Two-sided	Yes
3G	FT237 pscram-miR vs antimiR DO	Chi Square	0.1480, 1	0.3848	0.7004	ns	Two-sided	No
3G	FT237 pscram-miR vs pmiR-181a BR	Chi Square	4.082, 1	2.02	0.0434	*	Two-sided	Yes
3G	FT237 pscram-miR vs antimiR BR	Chi Square	NA	NA	NA	NA	NA	NA
3G	FT237 pscram-miR vs pmiR-181a DR	Chi Square	NA	NA	NA	NA	NA	NA
3G	FT237 pscram-miR vs antimiR DR	Chi Square	6.105, 1	2.471	0.0135	*	Two-sided	Yes

Supplemental Table 6: Figure 4 Statistical Tests

Figure Panel	Groups Compared	Statistical Test Used	P value	P value summary	Significantly different (P < 0.05)?	One- or two-tailed P value?	t, df	Difference of Means 95% Confidence Interval
4C	FT237 pscram-miR vs pmiR-181a	Unpaired t test	<0.0001	****	Yes	Two-tailed	t=9.091, df=78	32.57 to 50.84
4C	FT237 pscram-miR vs antimiR	Unpaired t test	0.3171	ns	No	Two-tailed	t=1.007, df=74	-2.275 to 6.926
4C	FT237 pmiR-181a vs antimiR	Unpaired t test	<0.0001	****	Yes	Two-tailed	t=8.183, df=76	-48.96 to -29.80

Supplemental Table 7: Figure 6 Statistical Tests

Figure Panel	Groups Compared	Statistical Test Used	P value	P value summary	Significantly different (P < 0.05)?	One- or two-tailed P value?	t, df	Difference of Means 95% Confidence Interval	
6B	FT237 pscram-miR vs pmiR-181a RNA	Unpaired t test	<0.0001	****	Yes	Two-tailed	t=15.87, df=4	-0.5993 to -0.4207	
6B	FT237 pscram-miR vs antimiR RNA	Unpaired t test	0.9365	ns	No	Two-tailed	t=0.08482, df=4	-0.1058 to 0.1124	
6B	FT237 pmiR-181a vs antimiR RNA	Unpaired t test	0.0005	***	Yes	Two-tailed	t=10.11, df=4	0.3724 to 0.6543	
6B	FT237 pscram-miR vs pmiR-181a protein	Unpaired t test	0.0052	**	Yes	Two-tailed	t=5.543, df=4	-0.6604 to -0.2196	
6B	FT237 pscram-miR vs antimiR protein	Unpaired t test	0.4125	ns	No	Two-tailed	t=0.9139, df=4	-0.2649 to 0.5249	
6B	FT237 pmiR-181a vs antimiR protein	Unpaired t test	0.0249	*	Yes	Two-tailed	t=3.499, df=4	0.1177 to 1.022	
6C	FT237 pscram-miR vs pmiR-181a	Unpaired t test	0.0002	***	Yes	Two-tailed	t=13.93, df=4	-0.4757 to -0.3176	
6C	FT237 pscram-miR vs antimiR	Unpaired t test	0.6493	ns	No	Two-tailed	t=0.4907, df=4	-0.3551 to 0.2484	
6C	FT237 pmiR-181a vs antimiR	Unpaired t test	0.0378	*	Yes	Two-tailed	t=3.056, df=4	0.03140 to 0.6553	
6C	FT237 pscram-miR vs pmiR-181a mut	Unpaired t test	<0.0001	****	Yes	Two-tailed	t=31.83, df=4	0.3052 to 0.3635	
6C	FT237 pscram-miR vs antimiR mut	Unpaired t test	<0.0001	****	Yes	Two-tailed	t=22.95, df=4	0.7355 to 0.9380	
6C	FT237 pmiR-181a vs antimiR mut	Unpaired t test	0.0002	***	Yes	Two-tailed	t=13.24, df=4	0.3970 to 0.6078	
6D	FT237 pscram-miR vs pmiR-181a RNA	Unpaired t test	0.0021	**	Yes	Two-tailed	t=7.112, df=4	-0.7184 to -0.3150	
6D	FT237 pscram-miR vs shrb1 RNA	Unpaired t test	<0.0001	****	Yes	Two-tailed	t=28.38, df=4	-0.5782 to -0.4751	
6D	FT237 pmiR-181a vs shrb1 RNA	Unpaired t test	0.9003	ns	No	Two-tailed	t=0.1334, df=4	-0.2182 to 0.1982	
6D	FT237 pscram-miR vs pmiR-181a protein	Unpaired t test	<0.0001	****	Yes	Two-tailed	t=19.39, df=4	-0.7050 to -0.5284	
6D	FT237 pscram-miR vs shrb1 protein	Unpaired t test	0.0004	***	Yes	Two-tailed	t=10.78, df=4	-0.9138 to -0.5395	
6D	FT237 pmiR-181a vs shrb1 protein	Unpaired t test	0.214	ns	No	Two-tailed	t=1.476, df=4	-0.3169 to 0.09694	
6E	FT237 pscram-miR vs pmiR-181a	Unpaired t test	0.0003	***	Yes	Two-tailed	t=7.470, df=6	14.93 to 29.48	
6E	FT237 pscram-miR vs shrb1	Unpaired t test	<0.0001	****	Yes	Two-tailed	t=23.12, df=6	14.64 to 18.11	
6E	FT237 pmiR-181a vs shrb1	Unpaired t test	0.1049	ns	No	Two-tailed	t=1.909, df=6	-13.31 to 1.645	
6F	FT237 pscram-miR vs pmiR-181a g2m	Unpaired t test	0.0003	***	Yes	Two-tailed	t=11.90, df=4	9.959 to 16.02	
6F	FT237 pscram-miR vs shrb1 g2m	Unpaired t test	<0.0001	****	Yes	Two-tailed	t=16.10, df=4	8.782 to 12.44	
6F	FT237 pmiR-181a vs shrb1 g2m	Unpaired t test	0.0729	ns	No	Two-tailed	t=2.419, df=4	-5.109 to 0.3517	
6F	FT237 pscram-miR vs pmiR-181a >4N	Unpaired t test	0.0002	***	Yes	Two-tailed	t=12.37, df=4	13.04 to 20.59	
6F	FT237 pscram-miR vs shrb1 >4N	Unpaired t test	0.001	***	Yes	Two-tailed	t=8.617, df=4	5.997 to 11.70	
6F	FT237 pmiR-181a vs shrb1 >4N	Unpaired t test	0.0031	**	Yes	Two-tailed	t=6.373, df=4	-11.44 to -4.497	
**Figure Panel**	**Groups Compared**	**Statistical Test Used**	**P value**	**P value summary**	**Significantly different (P < 0.05)?**	**One- or two-tailed P value?**			
6G	FT237 pmiR-181a vs shrb1	Fisher's exact test	>0.9999	ns	Two-sided	No			
**Figure Panel**	**Groups Compared**	**Statistical Test Used**	**P value**	**Exact or approximate P value?**	**P value summary**	**Significantly different (P < 0.05)?**	**One- or two-tailed P value?**	**Sum of ranks in column A,B**	**Mann-Whitney U**
6H	FT237 pmiR-181a vs shrb1 wk2	Mann Whitney U	0.5196	Exact	ns	No	Two-tailed	275.5 , 390.5	139.5
6H	FT237 pmiR-181a vs shrb1 wk4	Mann Whitney U	0.2071	Exact	ns	No	Two-tailed	256.5 , 409.5	120.5
6H	FT237 pmiR-181a vs shrb1 wk8	Mann Whitney U	0.721	Exact	ns	No	Two-tailed	290.5 , 304.5	133.5
6H	FT237 pmiR-181a vs shrb1 wk16	Mann Whitney U	0.0326	Exact	*	Yes	Two-tailed	175.5 , 352.5	70.5
6H	FT237 pmiR-181a vs shrb1 wk 34	Mann Whitney U	0.0889	Exact	ns	No	Two-tailed	302 , 259	88
6H	FT237 pmiR-181a vs shrb1 tb	Mann Whitney U	0.7005	Exact	ns	No	Two-tailed	220.5 , 307.5	115.5

Supplemental Table 8: Figure 7 Statistical Tests

Figure Panel	Groups Compared	Statistical Test Used	P value	P value summary	Significantly different (P < 0.05)?	One- or two-tailed P value?	t, df	Difference of Means 95% Confidence Interval
7A	FT237 pscram-miR vs pmiR-181a v	Unpaired t test	0.0011	**	Yes	Two-tailed	t=8.315, df=4	-0.4891 to -0.2442
7A	FT237 pscram-miR vs antimiR v	Unpaired t test	0.0004	***	Yes	Two-tailed	t=11.24, df=4	1.230 to 2.037
7A	FT237 pscram-miR vs shrb1 v	Unpaired t test	0.1583	ns	No	Two-tailed	t=1.732, df=4	-0.06030 to 0.2603
7A	FT237 pmiR-181a vs antimiR v	Unpaired t test	0.0002	***	Yes	Two-tailed	t=13.17, df=4	1.578 to 2.422
7A	FT237 pmiR-181a vs shrb1 v	Unpaired t test	0.003	**	Yes	Two-tailed	t=6.424, df=4	0.2650 to 0.6684
7A	FT237 pscram-miR vs pmiR-181a c	Unpaired t test	<0.0001	****	Yes	Two-tailed	t=22.37, df=4	-1.679 to -1.308
7A	FT237 pscram-miR vs antimiR c	Unpaired t test	<0.0001	****	Yes	Two-tailed	t=16.34, df=4	4.469 to 6.298
7A	FT237 pscram-miR vs shrb1 c	Unpaired t test	0.2152	ns	No	Two-tailed	t=1.471, df=4	-0.3176 to 0.09756
7A	FT237 pmiR-181a vs antimiR c	Unpaired t test	<0.0001	****	Yes	Two-tailed	t=21.07, df=4	5.971 to 7.783
7A	FT237 pmiR-181a vs shrb1 c	Unpaired t test	<0.0001	****	Yes	Two-tailed	t=23.20, df=4	1.218 to 1.549
7B	FT237 pscram-miR vs pmiR-181a v	Unpaired t test	<0.0001	****	Yes	Two-tailed	t=16.69, df=4	-0.7893 to -0.5641
7B	FT237 pscram-miR vs antimiR v	Unpaired t test	0.0094	**	Yes	Two-tailed	t=4.684, df=4	0.3176 to 1.242
7B	FT237 pmiR-181a vs antimiR v	Unpaired t test	0.0011	**	Yes	Two-tailed	t=8.499, df=4	0.9808 to 1.933
7B	FT237 pscram-miR vs pmiR-181a c	Unpaired t test	<0.0001	****	Yes	Two-tailed	t=19.56, df=4	-2.916 to -2.191
7B	FT237 pscram-miR vs antimiR c	Unpaired t test	0.0568	ns	No	Two-tailed	t=2.652, df=4	-0.06093 to 2.668
7B	FT237 pmiR-181a vs antimiR c	Unpaired t test	0.0013	**	Yes	Two-tailed	t=7.972, df=4	2.514 to 5.200
7C	FT237 pscram-miR vs pmiR-181a	Unpaired t test	0.0022	**	Yes	Two-tailed	t=7.000, df=4	-0.6518 to -0.2816
7C	FT237 pscram-miR vs antimiR	Unpaired t test	0.0029	**	Yes	Two-tailed	t=6.500, df=4	-0.2474 to -0.09929
7C	FT237 pmiR-181a vs antimiR	Unpaired t test	0.015	*	Yes	Two-tailed	t=4.085, df=4	0.09398 to 0.4927
7C	FT237 pscram-miR vs pmiR-181a mut	Unpaired t test	<0.0001	****	Yes	Two-tailed	t=24.84, df=4	0.4863 to 0.6088
7C	FT237 pscram-miR vs antimiR mut	Unpaired t test	0.001	***	Yes	Two-tailed	t=8.722, df=4	0.7316 to 1.415
7C	FT237 pmiR-181a vs antimiR mut	Unpaired t test	0.0136	*	Yes	Two-tailed	t=4.205, df=4	0.1785 to 0.8726
7D	FT237 pscram-miR vs pmiR-181a 2.5	Unpaired t test	<0.0001	****	Yes	Two-tailed	t=24.94, df=4	0.3004 to 0.3757
7D	FT237 pscram-miR vs antimiR 2.5	Unpaired t test	0.0413	*	Yes	Two-tailed	t=2.967, df=4	0.006561 to 0.1980
7D	FT237 pscram-miR vs shrb1 2.5	Unpaired t test	0.0882	ns	No	Two-tailed	t=2.244, df=4	-0.02426 to 0.2289
7D	FT237 pscram-miR vs pmiR-181a 5	Unpaired t test	<0.0001	****	Yes	Two-tailed	t=17.05, df=4	0.2713 to 0.3768
7D	FT237 pscram-miR vs antimiR 5	Unpaired t test	0.0039	**	Yes	Two-tailed	t=5.979, df=4	0.07684 to 0.2101
7D	FT237 pscram-miR vs shrb1 5	Unpaired t test	0.3508	ns	No	Two-tailed	t=1.055, df=4	-0.08661 to 0.1928
7D	FT237 pscram-miR vs pmiR-181a 10	Unpaired t test	0.0004	***	Yes	Two-tailed	t=10.97, df=4	0.2420 to 0.4059
7D	FT237 pscram-miR vs antimiR 10	Unpaired t test	0.0329	*	Yes	Two-tailed	t=3.199, df=4	0.007599 to 0.1076
7D	FT237 pscram-miR vs shrb1 10	Unpaired t test	0.0564	ns	No	Two-tailed	t=2.660, df=4	-0.002266 to 0.1061
7D	FT237 pscram-miR vs pmiR-181a 20	Unpaired t test	0.0006	***	Yes	Two-tailed	t=10.05, df=4	0.3131 to 0.5523
7D	FT237 pscram-miR vs antimiR 20	Unpaired t test	0.0675	ns	No	Two-tailed	t=2.490, df=4	-0.01029 to 0.1890
7D	FT237 pscram-miR vs shrb1 20	Unpaired t test	0.0819	ns	No	Two-tailed	t=2.311, df=4	-0.01881 to 0.2057
7E	FT237 pscram-miR v vs pscram-miR c	Unpaired t test	0.0006	***	Yes	Two-tailed	t=9.799, df=4	22.69 to 40.64
7E	FT237 pscram-miR v vs pmiR-181a v	Unpaired t test	0.0249	*	Yes	Two-tailed	t=3.500, df=4	-4.184 to -0.4824
7E	FT237 pscram-miR v vs pmiR-181a c	Unpaired t test	0.0002	***	Yes	Two-tailed	t=12.97, df=4	10.74 to 16.59
7E	FT237 pscram-miR v vs antimiR v	Unpaired t test	0.3739	ns	No	Two-tailed	t=1.000, df=4	-1.184 to 2.518
7E	FT237 pscram-miR v vs antimiR c	Unpaired t test	0.0042	**	Yes	Two-tailed	t=5.860, df=4	17.89 to 50.11
7E	FT237 pscram-miR c vs pmiR-181a c	Unpaired t test	0.0055	**	Yes	Two-tailed	t=5.455, df=4	-27.16 to -8.838
7E	FT237 pscram-miR c vs antimiR c	Unpaired t test	0.7412	ns	No	Two-tailed	t=0.3540, df=4	-15.97 to 20.63
7E	FT237 pmiR-181a c vs antimiR c	Unpaired t test	0.0253	*	Yes	Two-tailed	t=3.481, df=4	4.118 to 36.55
7F	FT237 pscram-miR vs pmiR-181a	Unpaired t test	0.0068	**	Yes	Two-tailed	t=5.142, df=4	-0.8862 to -0.2647
7F	FT237 pscram-miR vs shst1	Unpaired t test	0.0046	**	Yes	Two-tailed	t=5.740, df=4	-0.8395 to -0.2921
7F	FT237 pscram-miR vs shst2	Unpaired t test	<0.0001	****	Yes	Two-tailed	t=49.92, df=4	-0.9544 to -0.8538
7F	FT237 pmiR-181a vs shst1	Unpaired t test	0.9515	ns	No	Two-tailed	t=0.06479, df=4	-0.4044 to 0.4237
7F	FT237 pmiR-181a vs shst2	Unpaired t test	0.0442	*	Yes	Two-tailed	t=2.899, df=4	-0.6434 to -0.01390

Supplemental Table 9: Figure 7 Statistical Tests continued

Figure Panel	Groups Compared	Statistical Test Used	P value	P value summary	Significantly different (P < 0.05)?	One- or two-tailed P value?	t, df	Difference of Means 95% Confidence Interval
7G	FT237 pscram-miR v vs pscram-miR c	Unpaired t test	<0.0001	****	Yes	Two-tailed	t=26.63, df=4	-0.6679 to -0.5418
7G	FT237 pmiR-181a v vs miR-181a c	Unpaired t test	0.3972	ns	No	Two-tailed	t=0.9472, df=4	-0.1630 to 0.08006
7G	FT237 shst1 v vs shst1 c	Unpaired t test	0.2576	ns	No	Two-tailed	t=1.319, df=4	-0.2126 to 0.07563
7G	FT237 shst2 v vs shst2 c	Unpaired t test	0.0093	**	Yes	Two-tailed	t=4.708, df=4	-0.2121 to -0.05473
7G	FT237 pscram-miR vs pmiR-181a d2	Unpaired t test	<0.0001	****	Yes	Two-tailed	t=31.31, df=4	2.857 to 3.412
7G	FT237 pscram-miR vs shst1 d2	Unpaired t test	0.0005	***	Yes	Two-tailed	t=10.31, df=4	1.514 to 2.631
7G	FT237 pscram-miR vs shst2 d2	Unpaired t test	<0.0001	****	Yes	Two-tailed	t=19.15, df=4	1.176 to 1.574
7G	FT237 pscram-miR vs pmiR-181a d4	Unpaired t test	<0.0001	****	Yes	Two-tailed	t=19.89, df=4	5.529 to 7.323
7G	FT237 pscram-miR vs shst1 d4	Unpaired t test	0.0003	***	Yes	Two-tailed	t=12.12, df=4	4.853 to 7.737
7G	FT237 pscram-miR vs shst2 d4	Unpaired t test	0.0016	**	Yes	Two-tailed	t=7.566, df=4	3.735 to 8.066
7G	FT237 pscram-miR vs pmiR-181a d8	Unpaired t test	0.0016	**	Yes	Two-tailed	t=7.598, df=4	7.641 to 16.44
7G	FT237 pscram-miR vs shst1 d8	Unpaired t test	0.0005	***	Yes	Two-tailed	t=10.21, df=4	10.20 to 17.81
7G	FT237 pscram-miR vs shst2 d8	Unpaired t test	<0.0001	****	Yes	Two-tailed	t=19.92, df=4	15.66 to 20.73
7H	FT237 pscram-miR vs pmiR-181a	Unpaired t test	0.0099	**	Yes	Two-tailed	t=4.623, df=4	0.6589 to 2.640
7H	FT237 pscram-miR vs shst1	Unpaired t test	0.0055	**	Yes	Two-tailed	t=5.444, df=4	0.7209 to 2.221
7H	FT237 pscram-miR vs shst2	Unpaired t test	0.0007	***	Yes	Two-tailed	t=9.379, df=4	1.144 to 2.106
7H	FT237 pmiR-181a vs shst1	Unpaired t test	0.7105	ns	No	Two-tailed	t=0.3986, df=4	-1.421 to 1.064
7H	FT237 pmiR-181a vs shst2	Unpaired t test	0.9528	ns	No	Two-tailed	t=0.06299, df=4	-1.126 to 1.076
7I	FT237 pscram-miR vs pmiR-181a	Unpaired t test	0.0009	***	Yes	Two-tailed	t=8.912, df=4	12.05 to 22.95
7I	FT237 pscram-miR vs shst1	Unpaired t test	0.0107	*	Yes	Two-tailed	t=4.513, df=4	1.947 to 8.172
7I	FT237 pscram-miR vs shst2	Unpaired t test	0.0084	**	Yes	Two-tailed	t=4.844, df=4	6.351 to 23.41
7I	FT237 pmiR-181a vs shst1	Unpaired t test	0.0053	**	Yes	Two-tailed	t=5.504, df=4	-18.71 to -6.163
7I	FT237 pmiR-181a vs shst2	Unpaired t test	0.5125	ns	No	Two-tailed	t=0.7179, df=4	-12.74 to 7.503
7K	FT237 pscram-miR vs pmiR-181a g2m	Unpaired t test	0.0001	***	Yes	Two-tailed	t=15.12, df=4	11.95 to 17.32
7K	FT237 pscram-miR vs shst1 g2m	Unpaired t test	0.0001	***	Yes	Two-tailed	t=14.97, df=4	11.10 to 16.16
7K	FT237 pscram-miR vs shst2 g2m	Unpaired t test	0.0005	***	Yes	Two-tailed	t=10.37, df=4	7.742 to 13.40
7K	FT237 pmiR-181a vs shst1 g2m	Unpaired t test	0.1134	ns	No	Two-tailed	t=2.021, df=4	-2.382 to 0.3752
7K	FT237 pmiR-181a vs shst2 g2m	Unpaired t test	0.0039	**	Yes	Two-tailed	t=6.008, df=4	-5.940 to -2.185
7K	FT237 pscram-miR vs pmiR-181a >4n	Unpaired t test	0.023	*	Yes	Two-tailed	t=3.588, df=4	1.843 to 14.45
7K	FT237 pscram-miR vs shst1 >4n	Unpaired t test	0.0034	**	Yes	Two-tailed	t=6.227, df=4	2.859 to 7.460
7K	FT237 pscram-miR vs shst2 >4n	Unpaired t test	0.0019	**	Yes	Two-tailed	t=7.254, df=4	3.205 to 7.180
7K	FT237 pmiR-181a vs shst1 >4n	Unpaired t test	0.2538	ns	No	Two-tailed	t=1.332, df=4	-9.215 to 3.241
7K	FT237 pmiR-181a vs shst2 >4n	Unpaired t test	0.2512	ns	No	Two-tailed	t=1.340, df=4	-9.073 to 3.165
7L	FT237 pscram-miR vs pmiR-181a	Unpaired t test	0.0014	**	Yes	Two-tailed	t=7.869, df=4	11.86 to 24.79
7L	FT237 pscram-miR vs shst1	Unpaired t test	0.0004	***	Yes	Two-tailed	t=10.99, df=4	21.53 to 36.08
7L	FT237 pscram-miR vs shst2	Unpaired t test	0.0001	***	Yes	Two-tailed	t=15.29, df=4	35.26 to 50.91
7L	FT237 pmiR-181a vs shst1	Unpaired t test	0.0347	*	Yes	Two-tailed	t=3.145, df=4	1.228 to 19.72
7L	FT237 pmiR-181a vs shst2	Unpaired t test	0.0021	**	Yes	Two-tailed	t=7.095, df=4	15.07 to 34.44
7M	FT237 pscram-miR vs pmiR-181a	Unpaired t test	0.005	**	Yes	Two-tailed	t=5.588, df=4	0.7471 to 2.223
7M	FT237 pscram-miR vs shst1	Unpaired t test	0.0063	**	Yes	Two-tailed	t=5.245, df=4	0.7803 to 2.535
7M	FT237 pscram-miR vs shst2	Unpaired t test	0.0112	*	Yes	Two-tailed	t=4.456, df=4	1.446 to 6.228
7M	FT237 pmiR-181a vs shst1	Unpaired t test	0.6969	ns	No	Two-tailed	t=0.4188, df=4	-0.9735 to 1.319
7M	FT237 pmiR-181a vs shst2	Unpaired t test	0.0594	ns	No	Two-tailed	t=2.610, df=4	-0.1502 to 4.854

Supplemental Table 10: Figure 8 Statistical Tests

Figure Panel	Groups Compared	Statistical Test Used	P value	Exact or approximate P value?	P value summary	Significantly different (P < 0.05)?	One- or two-tailed P value?	Sum of ranks in column A,B	Mann-Whitney U
8B	181a high vs 181a low	Mann Whitney U test	0.0024	Approximate	**	Yes	Two-tailed	18297 , 14344	6343
8C	181a high vs 181a low	Mann Whitney U test	0.0033	Approximate	**	Yes	Two-tailed	22947 , 18381	8228
8D	181a high vs 181a low	Mann Whitney U test	0.0007	Approximate	***	Yes	Two-tailed	18502 , 14138	6137
8E	181a high vs 181a low	Mann Whitney U test	0.0361	Approximate	*	Yes	Two-tailed	22353 , 18975	8822

Supplemental Table 11: Supplemental Figure 1 Statistical Tests

Figure Panel	Groups Compared	Statistical Test Used	P value	P value summary	Significantly different (P < 0.05)?	One- or two-tailed P value?	t, df	Difference of Means 95% Confidence Interval
S1B	FT237 pscram-miR vs pmiR-181a	Unpaired t test	<0.0001	****	Yes	Two-tailed	t=17.24, df=4	19.97 to 27.65
S1B	FT237 pscram-miR vs antimiR	Unpaired t test	0.0003	***	Yes	Two-tailed	t=11.36, df=4	7.304 to 12.03
S1B	FT237 pmiR-181a vs antimiR	Unpaired t test	0.001	***	Yes	Two-tailed	t=8.716, df=4	-18.65 to -9.638
S1D	FT237 pscram-miR vs pmiR-181a d4	Unpaired t test	0.0002	***	Yes	Two-tailed	t=12.96, df=4	1.327 to 2.051
S1D	FT237 pscram-miR vs antimiR d4	Unpaired t test	0.0003	***	Yes	Two-tailed	t=12.07, df=4	1.799 to 2.874
S1D	FT237 pscram-miR vs pmiR-181a d8	Unpaired t test	<0.0001	****	Yes	Two-tailed	t=16.55, df=4	6.081 to 8.532
S1D	FT237 pscram-miR vs antimiR d8	Unpaired t test	0.0672	ns	No	Two-tailed	t=2.494, df=4	-0.1395 to 2.604
S1D	FT237 pscram-miR vs pmiR-181a d10	Unpaired t test	0.0003	***	Yes	Two-tailed	t=12.30, df=4	7.803 to 12.35
S1D	FT237 pscram-miR vs antimiR d10	Unpaired t test	0.0042	**	Yes	Two-tailed	t=5.860, df=4	2.066 to 5.786
S1D	FT237 pmiR-181a vs antimiR d10	Unpaired t test	0.0021	**	Yes	Two-tailed	t=7.058, df=4	-8.572 to -3.732
S1E	FT237 pscram-miR vs pmiR-181a	Unpaired t test	0.0002	***	Yes	Two-tailed	t=13.34, df=4	89.74 to 136.9
S1E	FT237 pscram-miR vs antimiR	Unpaired t test	0.0022	**	Yes	Two-tailed	t=6.978, df=4	35.93 to 83.41
S1E	FT237 pmiR-181a vs antimiR	Unpaired t test	0.0095	**	Yes	Two-tailed	t=4.675, df=4	-85.54 to -21.79
S1F	FT237 pscram-miR vs pmiR-181a	Unpaired t test	0.0002	***	Yes	Two-tailed	t=12.45, df=4	13.51 to 21.27
S1F	FT237 pscram-miR vs antimiR	Unpaired t test	0.0002	***	Yes	Two-tailed	t=13.59, df=4	2.888 to 4.371
S1F	FT237 pmiR-181a vs antimiR	Unpaired t test	0.0006	***	Yes	Two-tailed	t=9.699, df=4	-17.70 to -9.820
S1H	FT237 pscram-miR vs pmiR-181a g2m	Unpaired t test	<0.0001	****	Yes	Two-tailed	t=16.82, df=4	12.87 to 17.96
S1H	FT237 pscram-miR vs antimiR g2m	Unpaired t test	0.0024	**	Yes	Two-tailed	t=6.796, df=4	3.151 to 7.503
S1H	FT237 pmiR-181a vs antimiR g2m	Unpaired t test	0.0002	***	Yes	Two-tailed	t=12.57, df=4	-12.31 to -7.858
S1H	FT237 pscram-miR vs pmiR-181a >4n	Unpaired t test	0.0007	***	Yes	Two-tailed	t=9.353, df=4	4.217 to 7.778
S1H	FT237 pscram-miR vs antimiR >4n	Unpaired t test	0.0623	ns	No	Two-tailed	t=2.564, df=4	-2.545 to 0.1010
S1H	FT237 pmiR-181a vs antimiR >4n	Unpaired t test	0.0004	***	Yes	Two-tailed	t=11.14, df=4	-9.019 to -5.419

Supplemental Table 12: Supplemental Figure 2 Statistical Tests

Figure Panel	Groups Compared	Statistical Test Used	P value	P value summary	Significantly different (P < 0.05)?	One- or two-tailed P value?	t, df	Difference of Means 95% Confidence Interval
S2A	FT237 pscram-miR vs pmiR-181a an	Unpaired t test	0.0001	***	Yes	Two-tailed	t=14.94, df=4	49.48 to 72.07
S2A	FT237 pscram-miR vs antimiR an	Unpaired t test	0.0075	**	Yes	Two-tailed	t=5.002, df=4	5.085 to 17.77
S2A	FT237 pmiR-181a vs antimiR an	Unpaired t test	0.0001	***	Yes	Two-tailed	t=14.52, df=4	-58.78 to -39.91
S2A	FT237 pscram-miR vs pmiR-181a mn	Unpaired t test	0.0011	**	Yes	Two-tailed	t=8.363, df=4	11.43 to 22.80
S2A	FT237 pscram-miR vs antimiR mn	Unpaired t test	0.0369	*	Yes	Two-tailed	t=3.080, df=4	0.4362 to 8.414
S2A	FT237 pmiR-181a vs antimiR mn	Unpaired t test	0.004	**	Yes	Two-tailed	t=5.958, df=4	-18.61 to -6.777
S2A	FT237 pscram-miR vs pmiR-181a ml	Unpaired t test	0.0011	**	Yes	Two-tailed	t=8.456, df=4	20.01 to 39.58
S2A	FT237 pscram-miR vs antimiR ml	Unpaired t test	0.5771	ns	No	Two-tailed	t=0.6061, df=4	-3.743 to 5.833
S2A	FT237 pmiR-181a vs antimiR ml	Unpaired t test	0.0016	**	Yes	Two-tailed	t=7.600, df=4	-39.26 to -18.25
S2A	FT237 pscram-miR vs pmiR-181a bp	Unpaired t test	0.0277	*	Yes	Two-tailed	t=3.385, df=4	1.912 to 19.35
S2A	FT237 pscram-miR vs antimiR bp	Unpaired t test	0.8722	ns	No	Two-tailed	t=0.1715, df=4	-3.183 to 2.813
S2A	FT237 pmiR-181a vs antimiR bp	Unpaired t test	0.0285	*	Yes	Two-tailed	t=3.352, df=4	-19.78 to -1.858
S2B	FT240 pscram-miR vs pmiR-181a 1-0.8	Unpaired t test	0.0071	**	Yes	Two-tailed	t=5.083, df=4	-42.54 to -12.48
S2B	FT246 pscram-miR vs pmiR-181a 1-0.8	Unpaired t test	<0.0001	****	Yes	Two-tailed	t=22.23, df=4	-71.52 to -55.64
S2B	FT240 pscram-miR vs pmiR-181a 0.8-0.6	Unpaired t test	0.0378	*	Yes	Two-tailed	t=3.057, df=4	1.843 to 38.34
S2B	FT246 pscram-miR vs pmiR-181a 0.8-0.6	Unpaired t test	0.0003	***	Yes	Two-tailed	t=11.30, df=4	32.33 to 53.39
S2B	FT240 pscram-miR vs pmiR-181a 0.6-0.3	Unpaired t test	0.0311	*	Yes	Two-tailed	t=3.261, df=4	1.102 to 13.74
S2B	FT246 pscram-miR vs pmiR-181a 0.6-0.3	Unpaired t test	0.0005	***	Yes	Two-tailed	t=10.37, df=4	15.17 to 26.26

Supplemental Table 13: Supplemental Figure 3 Statistical Tests

Figure Panel	Groups Compared	Statistical Test Used	Chi-square, df	z	P value	P value summary	One- or two-sided	Statistically significant (P < 0.05)?
S3A	FT237 pscram-miR vs pmiR-181a B	Chi Square	49.62, 1	7.044	<0.0001	****	Two-sided	Yes
S3A	FT237 pscram-miR vs antimiR B	Chi Square	0.000, 1	0	>0.9999	ns	Two-sided	No
S3A	FT237 pscram-miR vs pmiR-181a G	Chi Square	97.20, 1	9.859	<0.0001	****	Two-sided	Yes
S3A	FT237 pscram-miR vs antimiR G	Chi Square	25.60, 1	5.06	<0.0001	****	Two-sided	Yes
S3A	FT237 pscram-miR vs pmiR-181a Y	Chi Square	8.292, 1	2.88	0.004	**	Two-sided	Yes
S3A	FT237 pscram-miR vs antimiR Y	Chi Square	19.64, 1	4.432	<0.0001	****	Two-sided	Yes
S3A	FT237 pscram-miR vs pmiR-181a R	Chi Square	67.01, 1	8.186	<0.0001	****	Two-sided	Yes
S3A	FT237 pscram-miR vs antimiR R	Chi Square	4.138, 1	2.034	0.0419	*	Two-sided	Yes

Figure Panel	Groups Compared	Statistical Test Used	P value	P value summary	Significantly different (P < 0.05)?	One- or two-tailed P value?	t, df	Difference of Means 95% Confidence Interval
S3B	FT237 pscram-miR vs pmiR-181a	Unpaired t test	<0.0001	****	Yes	Two-tailed	t=32.98, df=4	18.10 to 21.43
S3B	FT237 pscram-miR vs antimiR	Unpaired t test	0.0012	**	Yes	Two-tailed	t=8.259, df=4	5.489 to 11.05
S3B	FT237 pmiR-181a vs antimiR	Unpaired t test	0.0003	***	Yes	Two-tailed	t=11.45, df=4	-14.29 to -8.712
S3B	FT240 pscram-miR vs pmiR-181a	Unpaired t test	0.0043	**	Yes	Two-tailed	t=5.831, df=4	6.115 to 17.23
S3B	FT246 pscram-miR vs pmiR-181a	Unpaired t test	0.0035	**	Yes	Two-tailed	t=6.178, df=4	6.682 to 17.59

Supplemental Table 14: Supplemental Figure 3 Statistical Tests

Figure Panel	Groups Compared	Statistical Test Used	Chi-square, df	z	P value	P value summary	One- or two-sided	Statistically significant (P < 0.05)?
S4B	FT237 pscram-miR vs pmiR-181a B	Chi Square	3.920, 1	1.98	0.0477	*	Two-sided	Yes
S4B	FT237 pscram-miR vs antimiR B	Chi Square	3.875, 1	1.969	0.049	*	Two-sided	Yes
S4C	FT237 pscram-miR vs pmiR-181a p	Chi Square	13.53, 1	3.679	0.0002	***	Two-sided	Yes
S4C	FT237 pscram-miR vs antimiR p	Chi Square	1.220, 1	1.105	0.2693	ns	Two-sided	No
S4C	FT237 pscram-miR vs pmiR-181a db	Chi Square	0.001209, 1	0.03477	0.9723	ns	Two-sided	No
S4C	FT237 pscram-miR vs antimiR db	Chi Square	1.057, 1	1.028	0.3039	ns	Two-sided	No
S4C	FT237 pscram-miR vs pmiR-181a mb	Chi Square	2.141, 1	1.463	0.1434	ns	Two-sided	No
S4C	FT237 pscram-miR vs antimiR mb	Chi Square	NA					
S4C	FT237 pscram-miR vs pmiR-181a lb	Chi Square	14.92, 1	3.863	0.0001	***	Two-sided	Yes
S4C	FT237 pscram-miR vs antimiR lb	Chi Square	NA					
S4C	FT237 pscram-miR vs pmiR-181a g	Chi Square	1.567, 1	1.252	0.2106	ns	Two-sided	No
S4C	FT237 pscram-miR vs antimiR g	Chi Square	5.032, 1	2.243	0.0249	*	Two-sided	Yes
S4C	FT237 pscram-miR vs pmiR-181a o	Chi Square	0.001209, 1	0.03477	0.9723	ns	Two-sided	No
S4C	FT237 pscram-miR vs antimiR o	Chi Square	2.301, 1	1.517	0.1293	ns	Two-sided	No
S4C	FT237 pscram-miR vs pmiR-181a r	Chi Square	2.005, 1	1.416	0.1568	ns	Two-sided	No
S4C	FT237 pscram-miR vs antimiR r	Chi Square	2.954, 1	1.719	0.0857	ns	Two-sided	No

Supplemental Table 15: Supplemental Figure 6 Statistical Tests

Figure Panel	Groups Compared	Statistical Test Used	P value	P value summary	Significantly different (P < 0.05)?	One- or two-tailed P value?	t, df	Difference of Means 95% Confidence Interval
S6A	FT240 pscram-miR vs pmiR-181a	Unpaired t test	0.0012	**	Yes	Two-tailed	t=8.188, df=4	-0.6963 to -0.3437
S6A	FT246 pscram-miR vs pmiR-181a	Unpaired t test	<0.0001	****	Yes	Two-tailed	t=29.43, df=4	-0.7515 to -0.6219
S6B	FT240 pscram-miR vs pmiR-181a	Unpaired t test	<0.0001	****	Yes	Two-tailed	t=30.90, df=4	-0.3003 to -0.2507
S6B	FT246 pscram-miR vs pmiR-181a	Unpaired t test	0.0004	***	Yes	Two-tailed	t=10.87, df=4	-0.4813 to -0.2854
S6C	FT240 pscram-miR vs pmiR-181a	Unpaired t test	0.0001	***	Yes	Two-tailed	t=15.30, df=4	-0.9412 to -0.6521
S6C	FT246 pscram-miR vs pmiR-181a	Unpaired t test	0.0014	**	Yes	Two-tailed	t=7.938, df=4	-0.8368 to -0.4032
S6D	FT240 pscram-miR vs pmiR-181a	Unpaired t test	<0.0001	****	Yes	Two-tailed	t=42.14, df=4	-0.6702 to -0.5874
S6D	FT246 pscram-miR vs pmiR-181a	Unpaired t test	0.0001	***	Yes	Two-tailed	t=14.14, df=4	-0.6859 to -0.4607

Supplemental Table 16: Supplemental Figure 7 Statistical Tests

Figure Panel	Groups Compared	Statistical Test Used	P value	P value summary	Significantly different (P < 0.05)?	One- or two-tailed P value?	t, df	Difference of Means 95% Confidence Interval
S7A	FT237 pscram-miR vs pmiR-181a d2	Unpaired t test	0.0002	***	Yes	Two-tailed	t=12.32, df=4	1.501 to 2.375
S7A	FT237 pscram-miR vs pshrb1 d2	Unpaired t test	<0.0001	****	Yes	Two-tailed	t=34.88, df=4	1.599 to 1.875
S7A	FT237 pscram-miR vs pmiR-181a d4	Unpaired t test	<0.0001	****	Yes	Two-tailed	t=45.80, df=4	3.248 to 3.667
S7A	FT237 pscram-miR vs pshrb1 d4	Unpaired t test	<0.0001	****	Yes	Two-tailed	t=89.28, df=4	4.070 to 4.332
S7A	FT237 pscram-miR vs pmiR-181a d6	Unpaired t test	<0.0001	****	Yes	Two-tailed	t=15.86, df=4	5.471 to 7.794
S7A	FT237 pscram-miR vs pshrb1 d6	Unpaired t test	<0.0001	****	Yes	Two-tailed	t=52.81, df=4	6.522 to 7.246
S7A	FT237 pscram-miR vs pmiR-181a d8	Unpaired t test	<0.0001	****	Yes	Two-tailed	t=25.76, df=4	11.06 to 13.73
S7A	FT237 pscram-miR vs pshrb1 d8	Unpaired t test	<0.0001	****	Yes	Two-tailed	t=80.52, df=4	12.01 to 12.87
S7A	FT237 pscram-miR vs pmiR-181a d10	Unpaired t test	<0.0001	****	Yes	Two-tailed	t=40.05, df=4	13.45 to 15.45
S7A	FT237 pscram-miR vs pshrb1 d10	Unpaired t test	<0.0001	****	Yes	Two-tailed	t=60.88, df=4	12.73 to 13.94
S7A	FT237 pmiR-181a vs pshrb1 d8	Unpaired t test	0.9207	ns	No	Two-tailed	t=0.1060, df=4	-1.343 to 1.450
S7A	FT237 pmiR-181a vs pshrb1 d10	Unpaired t test	0.0441	*	Yes	Two-tailed	t=2.900, df=4	-2.186 to -0.04770
S7B	FT237 pscram-miR vs pmiR-181a	Unpaired t test	<0.0001	****	Yes	Two-tailed	t=31.91, df=6	4.572 to 5.332
S7B	FT237 pscram-miR vs pshrb1	Unpaired t test	<0.0001	****	Yes	Two-tailed	t=43.41, df=6	4.946 to 5.536
S7B	FT237 pmiR-181a vs pshrb1	Unpaired t test	0.1922	ns	No	Two-tailed	t=1.469, df=6	-0.1923 to 0.7700
S7C	FT237 pscram-miR vs pmiR-181a 1-0.8	Unpaired t test	0.0005	***	Yes	Two-tailed	t=10.22, df=4	-87.35 to -50.02
S7C	FT237 pscram-miR vs pshrb1 1-0.8	Unpaired t test	0.0208	*	Yes	Two-tailed	t=3.704, df=4	-42.41 to -6.070
S7C	FT237 pmiR-181a vs pshrb1 1-0.8	Unpaired t test	0.0004	***	Yes	Two-tailed	t=10.70, df=4	32.91 to 55.98
S7C	FT237 pscram-miR vs pmiR-181a 0.8-0.6	Unpaired t test	0.001	**	Yes	Two-tailed	t=8.586, df=4	28.10 to 54.96
S7C	FT237 pscram-miR vs pshrb1 0.8-0.6	Unpaired t test	0.0403	*	Yes	Two-tailed	t=2.991, df=4	1.037 to 27.88
S7C	FT237 pmiR-181a vs pshrb1 0.8-0.6	Unpaired t test	0.0002	***	Yes	Two-tailed	t=13.15, df=4	-32.79 to -21.35
S7C	FT237 pscram-miR vs pmiR-181a 0.6-0.3	Unpaired t test	0.0007	***	Yes	Two-tailed	t=9.597, df=4	19.42 to 35.23
S7C	FT237 pscram-miR vs pshrb1 0.6-0.3	Unpaired t test	0.0222	*	Yes	Two-tailed	t=3.626, df=4	2.291 to 17.26
S7C	FT237 pmiR-181a vs pshrb1 0.6-0.3	Unpaired t test	0.0017	**	Yes	Two-tailed	t=7.481, df=4	-24.06 to -11.04
S7D	FT237 pscram-miR vs pmiR-181a	Unpaired t test	<0.0001	****	Yes	Two-tailed	t=16.54, df=4	22.70 to 31.85
S7D	FT237 pscram-miR vs pshrb1	Unpaired t test	0.0016	**	Yes	Two-tailed	t=7.598, df=4	11.35 to 24.43
S7D	FT237 pmiR-181a vs pshrb1	Unpaired t test	0.0169	*	Yes	Two-tailed	t=3.947, df=4	-15.99 to -2.784

Supplemental Table 17: Supplemental Figure 8 Statistical Tests

Figure Panel	Groups Compared	Statistical Test Used	P value	P value summary	Significantly different (P < 0.05)?	One- or two-tailed P value?	t, df	Difference of Means 95% Confidence Interval
S8A	FT194 pscram-miR vs pmiR-181a d2	Unpaired t test	0.9301	ns	No	Two-tailed	t=0.09334, df=4	-0.5462 to 0.5842
S8A	FT194 pscram-miR vs pmiR-181a d4	Unpaired t test	0.0823	ns	No	Two-tailed	t=2.307, df=4	-0.2335 to 2.526
S8A	FT194 pscram-miR vs pmiR-181a d6	Unpaired t test	0.1035	ns	No	Two-tailed	t=2.101, df=4	-0.4017 to 2.901
S8B	FT194 pscram-miR vs pmiR-181a 1-0.8	Unpaired t test	0.8342	ns	No	Two-tailed	t=0.2163, df=8	-11.42 to 13.79
S8B	FT194 pscram-miR vs pmiR-181a 0.8-0.6	Unpaired t test	0.5193	ns	No	Two-tailed	t=0.6741, df=8	-12.57 to 22.95
S8B	FT194 pscram-miR vs pmiR-181a 0.6-0.3	Unpaired t test	0.2934	ns	No	Two-tailed	t=1.124, df=8	-19.44 to 6.697
S8C	FT194 pscram-miR vs pmiR-181a	Unpaired t test	0.2442	ns	No	Two-tailed	t=1.364, df=4	-7.574 to 22.21
S8D	FT194 pscram-miR vs pmiR-181a	Unpaired t test	0.2014	ns	No	Two-tailed	t=1.527, df=4	-0.4978 to 0.1445
S8E	FT194 pscram-miR vs pmiR-181a g2m	Unpaired t test	0.6778	ns	No	Two-tailed	t=0.4474, df=4	-6.504 to 4.699
S8E	FT194 pscram-miR vs pmiR-181a >4n	Unpaired t test	0.8447	ns	No	Two-tailed	t=0.2089, df=4	-3.912 to 3.365

Supplemental Table 18: Supplemental Figure 9 Statistical Tests

Figure Panel	Groups Compared	Statistical Test Used	P value	P value summary	Significantly different (P < 0.05)?	One- or two-tailed P value?	t, df	Difference of Means 95% Confidence Interval
S9B	FT237 pscram-miR vs pmiR-181a v l	Unpaired t test	0.2063	ns	No	Two-tailed	t=1.507, df=4	-0.2686 to 0.9062
S9B	FT237 pscram-miR vs antimiR v l	Unpaired t test	<0.0001	****	Yes	Two-tailed	t=29.13, df=4	1.715 to 2.076
S9B	FT237 pmiR-181a vs antimiR v l	Unpaired t test	<0.0001	****	Yes	Two-tailed	t=27.21, df=4	1.616 to 1.983
S9B	FT237 pscram-miR vs pmiR-181a c l	Unpaired t test	0.0001	***	Yes	Two-tailed	t=15.23, df=4	-194.0 to -134.2
S9B	FT237 pscram-miR vs antimiR c l	Unpaired t test	0.0005	***	Yes	Two-tailed	t=10.57, df=4	-148.1 to -86.49
S9B	FT237 pmiR-181a vs antimiR c i	Unpaired t test	0.0003	***	Yes	Two-tailed	t=11.78, df=4	35.78 to 57.84
S9B	FT237 pscram-miR vs pmiR-181a v t	Unpaired t test	<0.0001	****	Yes	Two-tailed	t=29.34, df=4	-0.3300 to -0.2730
S9B	FT237 pscram-miR vs antimiR v t	Unpaired t test	<0.0001	****	Yes	Two-tailed	t=16.05, df=4	1.857 to 2.633
S9B	FT237 pmiR-181a vs antimiR v t	Unpaired t test	<0.0001	****	Yes	Two-tailed	t=18.16, df=4	2.157 to 2.936
S9B	FT237 pscram-miR vs pmiR-181a c t	Unpaired t test	<0.0001	****	Yes	Two-tailed	t=55.52, df=4	-17.76 to -16.07
S9B	FT237 pscram-miR vs antimiR c t	Unpaired t test	<0.0001	****	Yes	Two-tailed	t=32.62, df=4	-11.77 to -9.922
S9B	FT237 pmiR-181a vs antimiR c t	Unpaired t test	<0.0001	****	Yes	Two-tailed	t=18.09, df=4	5.137 to 7.000
S9B	FT237 pscram-miR vs pmiR-181a v cx	Unpaired t test	0.0026	**	Yes	Two-tailed	t=6.674, df=4	-0.5100 to -0.2103
S9B	FT237 pscram-miR vs antimiR v cx	Unpaired t test	0.1988	ns	No	Two-tailed	t=1.538, df=4	-0.4054 to 1.412
S9B	FT237 pmiR-181a vs antimiR v cx	Unpaired t test	0.0599	ns	No	Two-tailed	t=2.603, df=4	-0.05750 to 1.785
S9B	FT237 pscram-miR vs pmiR-181a c cx	Unpaired t test	0.0018	**	Yes	Two-tailed	t=7.340, df=4	-9086 to -4099
S9B	FT237 pscram-miR vs antimiR c cx	Unpaired t test	0.0027	**	Yes	Two-tailed	t=6.636, df=4	-8543 to -3503
S9B	FT237 pmiR-181a vs antimiR c cx	Unpaired t test	0.0198	*	Yes	Two-tailed	t=3.761, df=4	149.1 to 989.9
S9C	FT237 pscram-miR vs pmiR-181a c	Unpaired t test	0.0002	***	Yes	Two-tailed	t=12.55, df=4	-5460 to -3482
S9C	FT237 pscram-miR vs antimiR c	Unpaired t test	0.0002	***	Yes	Two-tailed	t=13.35, df=4	-2344 to -1537
S9C	FT237 pscram-miR vs shrb1 c	Unpaired t test	0.0004	***	Yes	Two-tailed	t=10.65, df=4	-1695 to -994.0
S9C	FT237 pmiR-181a vs antimiR c	Unpaired t test	0.002	**	Yes	Two-tailed	t=7.221, df=4	1558 to 3503
S9C	FT237 pmiR-181a vs shrb1 c	Unpaired t test	0.0008	***	Yes	Two-tailed	t=9.117, df=4	2174 to 4078
S9D	FT237 pscram-miR vs pmiR-181a	Unpaired t test	0.0025	**	Yes	Two-tailed	t=6.755, df=4	0.7347 to 1.760
S9D	FT237 pscram-miR vs shst1	Unpaired t test	0.0057	**	Yes	Two-tailed	t=5.401, df=4	0.5742 to 1.789
S9D	FT237 pscram-miR vs shst2	Unpaired t test	0.0046	**	Yes	Two-tailed	t=5.713, df=4	0.4438 to 1.283
S9D	FT237 pmiR-181a vs shst1	Unpaired t test	0.8292	ns	No	Two-tailed	t=0.2302, df=4	-0.8607 to 0.7289
S9D	FT237 pmiR-181a vs shst2	Unpaired t test	0.1828	ns	No	Two-tailed	t=1.609, df=4	-1.047 to 0.2785
S9D	FT237 pscram-miR vs pmiR-181a	Unpaired t test	0.0079	**	Yes	Two-tailed	t=4.922, df=4	0.6480 to 2.325
S9D	FT237 pscram-miR vs scram stoe	Unpaired t test	<0.0001	****	Yes	Two-tailed	t=34.83, df=4	-0.9789 to -0.8344
S9D	FT237 pscram-miR vs 181a stoe	Unpaired t test	0.6978	ns	No	Two-tailed	t=0.4174, df=4	-0.7142 to 0.5276
S9D	FT237 pmiR-181a vs scram stoe	Unpaired t test	<0.0001	****	Yes	Two-tailed	t=34.83, df=4	-0.9789 to -0.8344
S9D	FT237 pmiR-181a vs 181a stoe	Unpaired t test	0.6978	ns	No	Two-tailed	t=0.4174, df=4	-0.7142 to 0.5276
S9E	FT237 pscram-miR v vs pscam-miR c	Unpaired t test	0.0008	***	Yes	Two-tailed	t=9.171, df=4	6.740 to 12.59
S9E	FT237 pscram-miR v vs pmiR-181a v	Unpaired t test	0.1493	ns	No	Two-tailed	t=1.782, df=4	-5.116 to 1.116
S9E	FT237 pscram-miR v vs pmiR-181a c	Unpaired t test	0.121	ns	No	Two-tailed	t=1.964, df=4	-4.827 to 0.8274
S9E	FT237 pscram-miR v vs shst1 v	Unpaired t test	0.1354	ns	No	Two-tailed	t=1.867, df=4	-4.975 to 0.9750
S9E	FT237 pscram-miR v vs shst1 c	Unpaired t test	0.139	ns	No	Two-tailed	t=1.844, df=4	-4.733 to 0.9553
S9E	FT237 pscram-miR v vs shst2 v	Unpaired t test	0.1544	ns	No	Two-tailed	t=1.753, df=4	-4.880 to 1.102
S9E	FT237 pscram-miR v vs shst2 c	Unpaired t test	0.1951	ns	No	Two-tailed	t=1.554, df=4	-4.954 to 1.398
S9E	FT237 pscram-miR v vs scram stoe v	Unpaired t test	0.0022	**	Yes	Two-tailed	t=7.012, df=4	17.15 to 39.63
S9E	FT237 pscram-miR v vs scram stoe c	Unpaired t test	0.0001	***	Yes	Two-tailed	t=15.07, df=4	44.32 to 64.34
S9E	FT237 pscram-miR v vs p181a stoe v	Unpaired t test	0.0959	ns	No	Two-tailed	t=2.169, df=4	-5.066 to 0.6220
S9E	FT237 pscram-miR v vs 181a stoe c	Unpaired t test	0.072	ns	No	Two-tailed	t=2.429, df=4	-5.238 to 0.3491

Supplemental Table 19: Supplemental Figure 9 Statistical Tests cont

Figure Panel	Groups Compared	Statistical Test Used	P value	P value summary	Significantly different (P < 0.05)?	One- or two-tailed P value?	t, df	Difference of Means 95% Confidence Interval
S9E	FT237 pscram-miR c vs pmiR-181a c	Unpaired t test	<0.0001	****	Yes	Two-tailed	t=30.31, df=4	-12.74 to -10.60
S9E	FT237 pscram-miR c vs shst1 c	Unpaired t test	<0.0001	****	Yes	Two-tailed	t=28.84, df=4	-12.67 to -10.44
S9E	FT237 pscram-miR c vs shst2 c	Unpaired t test	<0.0001	****	Yes	Two-tailed	t=17.66, df=4	-13.24 to -9.646
S9E	FT237 pscram-miR c vs scram stoe c	Unpaired t test	0.0002	***	Yes	Two-tailed	t=12.83, df=4	35.00 to 54.33
S9E	FT237 pscram-miR c vs 181a stoe c	Unpaired t test	<0.0001	****	Yes	Two-tailed	t=34.47, df=4	-13.09 to -11.14
S9E	FT237 pscram-miR v vs pscram stoe c	Unpaired t test	0.0077	**	Yes	Two-tailed	t=4.957, df=4	11.41 to 40.48

Supplemental Table 20: Supplemental Figure 10 Statistical Tests

Figure Panel	Groups Compared	Statistical Test Used	P value	P value summary	Significantly different (P < 0.05)?	One- or two-tailed P value?	t, df	Difference of Means 95% Confidence Interval
S10A	FT237 pscram-miR vs pmiR-181a	Unpaired t test	0.0011	**	Yes	Two-tailed	t=8.390, df=4	-0.4370 to -0.2197
S10A	FT237 pscram-miR vs scram stoe	Unpaired t test	0.0013	**	Yes	Two-tailed	t=8.060, df=4	3.461 to 7.099
S10A	FT237 pscram-miR vs 181a stoe	Unpaired t test	0.0005	***	Yes	Two-tailed	t=10.55, df=4	4.076 to 6.988
S10A	FT237 pmiR-181a vs scram stoe	Unpaired t test	0.001	**	Yes	Two-tailed	t=8.546, df=4	3.787 to 7.431
S10A	FT237 pmiR-181a vs 181a stoe	Unpaired t test	0.0004	***	Yes	Two-tailed	t=11.15, df=4	4.401 to 7.320
S10B	FT237 pscram-miR v vs pscram-miR c	Unpaired t test	<0.0001	****	Yes	Two-tailed	t=55.98, df=4	-0.6130 to -0.5551
S10B	FT237 pmiR-181a v vs pmiR-181a c	Unpaired t test	0.0006	***	Yes	Two-tailed	t=9.910, df=4	-0.1974 to -0.1110
S10B	FT237 scram stoe v vs scram stoe c	Unpaired t test	<0.0001	****	Yes	Two-tailed	t=28.12, df=4	-0.9198 to -0.7545
S10B	FT237 181a stoe v vs 181a stoe c	Unpaired t test	0.0001	***	Yes	Two-tailed	t=14.46, df=4	-0.6209 to -0.4209
S10C	FT237 pscram-miR vs pmiR-181a d2	Unpaired t test	0.0003	***	Yes	Two-tailed	t=12.17, df=4	1.951 to 3.104
S10C	FT237 pscram-miR vs scram stoe d2	Unpaired t test	0.0088	**	Yes	Two-tailed	t=4.782, df=4	0.3819 to 1.439
S10C	FT237 pscram-miR vs 181a stoe d2	Unpaired t test	0.0001	***	Yes	Two-tailed	t=14.33, df=4	1.839 to 2.723
S10C	FT237 pscram-miR vs pmiR-181a d4	Unpaired t test	0.0001	***	Yes	Two-tailed	t=14.67, df=4	5.224 to 7.663
S10C	FT237 pscram-miR vs scram stoe d4	Unpaired t test	0.0109	*	Yes	Two-tailed	t=4.491, df=4	0.6191 to 2.624
S10C	FT237 pscram-miR vs 181a stoe d4	Unpaired t test	0.0003	***	Yes	Two-tailed	t=12.07, df=4	3.352 to 5.355
S10C	FT237 pscram-miR vs pmiR-181a d8	Unpaired t test	0.0012	**	Yes	Two-tailed	t=8.247, df=4	6.722 to 13.54
S10C	FT237 pscram-miR vs scram stoe d8	Unpaired t test	0.3325	ns	No	Two-tailed	t=1.102, df=4	-1.892 to 4.380
S10C	FT237 pscram-miR vs 181a stoe d8	Unpaired t test	0.0185	*	Yes	Two-tailed	t=3.840, df=4	1.519 to 9.450
S10D	FT237 pscram-miR vs pmiR-181a	Unpaired t test	0.0001	***	Yes	Two-tailed	t=15.12, df=4	14.70 to 21.30
S10D	FT237 pscram-miR vs scram stoe	Unpaired t test	0.1161	ns	No	Two-tailed	t=2.000, df=4	-1.592 to 0.2588
S10D	FT237 pscram-miR vs 181a stoe	Unpaired t test	0.0006	***	Yes	Two-tailed	t=10.00, df=4	4.816 to 8.518
S10D	FT237 pmiR-181a vs scram stoe	Unpaired t test	<0.0001	****	Yes	Two-tailed	t=16.00, df=4	-21.91 to -15.43
S10D	FT237 pmiR-181a vs 181a stoe	Unpaired t test	0.001	***	Yes	Two-tailed	t=8.707, df=4	-14.95 to -7.719
S10F	FT237 pscram-miR vs pmiR-181a g2m	Unpaired t test	<0.0001	****	Yes	Two-tailed	t=35.18, df=4	7.878 to 9.228
S10F	FT237 pscram-miR vs scram stoe g2m	Unpaired t test	<0.0001	****	Yes	Two-tailed	t=88.32, df=4	21.93 to 23.36
S10F	FT237 pscram-miR vs 181a stoe g2m	Unpaired t test	<0.0001	****	Yes	Two-tailed	t=76.95, df=4	30.88 to 33.19
S10F	FT237 pscram-miR vs pmiR-181a >4n	Unpaired t test	<0.0001	****	Yes	Two-tailed	t=22.86, df=4	8.651 to 11.04
S10F	FT237 pscram-miR vs scram stoe >4n	Unpaired t test	<0.0001	****	Yes	Two-tailed	t=27.57, df=4	13.25 to 16.22
S10F	FT237 pscram-miR vs 181a stoe >4n	Unpaired t test	<0.0001	****	Yes	Two-tailed	t=16.70, df=4	7.145 to 9.995
S10F	FT237 pscram-miR vs pmiR-181a sg1	Unpaired t test	0.0161	*	Yes	Two-tailed	t=4.002, df=4	0.3209 to 1.775
S10F	FT237 pscram-miR vs scram stoe sg1	Unpaired t test	0.0004	***	Yes	Two-tailed	t=10.93, df=4	1.707 to 2.869
S10F	FT237 pscram-miR vs 181a stoe sg1	Unpaired t test	0.0023	**	Yes	Two-tailed	t=6.913, df=4	1.943 to 4.551
S10F	FT237 pmiR-181a vs scram stoe sg1	Unpaired t test	0.0167	*	Yes	Two-tailed	t=3.961, df=4	0.3707 to 2.109
S10F	FT237 pmiR-181a vs 181a stoe sg1	Unpaired t test	0.0137	*	Yes	Two-tailed	t=4.195, df=4	0.7438 to 3.655
S10F	FT237 pscram-miR vs scram stoe sg1fc	Unpaired t test	0.0084	**	Yes	Two-tailed	t=4.844, df=4	1.453 to 5.355
S10F	FT237 pmiR-181a vs 181a stoe sg1fc	Unpaired t test	0.035	*	Yes	Two-tailed	t=3.136, df=4	0.1312 to 2.158
S10F	FT237 pscram-miR vs scram stoe g2mfc	Unpaired t test	<0.0001	****	Yes	Two-tailed	t=42.24, df=4	1.003 to 1.144
S10F	FT237 pmiR-181a vs 181a stoe g2mfc	Unpaired t test	<0.0001	****	Yes	Two-tailed	t=40.93, df=4	0.7453 to 0.8538
S10F	FT237 pscram-miR vs scram stoe >4nfc	Unpaired t test	<0.0001	****	Yes	Two-tailed	t=75.32, df=4	0.7920 to 0.8526
S10F	FT237 pmiR-181a vs 181a stoe >4nfc	Unpaired t test	<0.0001	****	Yes	Two-tailed	t=77.74, df=4	-2.007 to -1.868
S10G	FT237 pscram-miR vs pmiR-181a	Unpaired t test	<0.0001	****	Yes	Two-tailed	t=18.69, df=4	15.49 to 20.89
S10G	FT237 pscram-miR vs scram stoe	Unpaired t test	0.2818	ns	No	Two-tailed	t=1.243, df=4	-1.193 to 3.128
S10G	FT237 pscram-miR vs 181a stoe	Unpaired t test	0.1707	ns	No	Two-tailed	t=1.668, df=4	-1.845 to 7.396
S10G	FT237 pmiR-181a vs scram stoe	Unpaired t test	<0.0001	****	Yes	Two-tailed	t=23.39, df=4	-19.27 to -15.18
S10G	FT237 pmiR-181a vs 181a stoe	Unpaired t test	0.0007	***	Yes	Two-tailed	t=9.369, df=4	-19.98 to -10.85
S10H	FT237 pscram-miR vs pmiR-181a	Unpaired t test	0.0009	***	Yes	Two-tailed	t=8.868, df=4	0.5574 to 1.065
S10H	FT237 pscram-miR vs scram stoe	Unpaired t test	<0.0001	****	Yes	Two-tailed	t=22.07, df=4	-0.9118 to -0.7081
S10H	FT237 pscram-miR vs 181a stoe	Unpaired t test	<0.0001	****	Yes	Two-tailed	t=48.66, df=4	-0.6772 to -0.6041
S10H	FT237 pmiR-181a vs scram stoe	Unpaired t test	<0.0001	****	Yes	Two-tailed	t=16.45, df=4	-1.895 to -1.348
S10H	FT237 pmiR-181a vs 181a stoe	Unpaired t test	<0.0001	****	Yes	Two-tailed	t=15.71, df=4	-1.709 to -1.195
