# Supplementary material for: miR-181a initiates and perpetuates oncogenic transformation through the regulation of innate immune signaling
Source: Nat Commun. 2020 Jun 26;11:3231. doi: 10.1038/s41467-020-17030-w (PMC7320168; doi:10.1038/s41467-020-17030-w)
Supplement: Supplementary file 3 — Description of Additional Supplementary Files [file 41467_2020_17030_MOESM3_ESM.pdf]

Description of Additional Supplementary Files

File Name: Supplementary Movie 1A
Description: FT237 pscram-miR SV40-GFP cell division

File Name: Supplementary Movie 1B
Description: FT237 pmiR-181a SV40-GFP cell division

File Name: Supplementary Movie 1C
Description: FT237 pmiR-181a + antimiR SV40-GFP cell division

File Name: Supplementary Movie 2A
Description: FT237 pscram-miR nucleus rupture SV40 GFP

File Name: Supplementary Movie 2B
Description: FT237 pscram-miR nucleus rupture MERGE

File Name: Supplementary Movie 2C
Description: FT237 pmiR-181a nucleus rupture SV40 GFP

File Name: Supplementary Movie 2D
Description: FT237 pmiR-181a nucleus rupture MERGE

File Name: Supplementary Movie 3A
Description: FT237 pmiR-181a nucleoplasmic bridge H2B-GFP

File Name: Supplementary Movie 3B
Description: FT237 pmiR-181a nucleoplasmic bridge merge

File Name: Supplementary Movie 3C
Description: FT237 pmiR-181a lagging chromosome H2B-GFP

File Name: Supplementary Movie 3D
Description: FT237 pmiR-181a lagging chromosome merge

File Name: Supplementary Movie 3E
Description: FT237 pmiR-181a failed cytokinesis H2B-GFP

File Name: Supplementary Movie 3F
Description: FT237 pmiR-181a failed cytokinesis merge

File Name: Supplementary Movie 3G
Description: FT237 pmiR-181a multipolar cytokinesis H2B-GFP

File Name: Supplementary Movie 3H
Description: FT237 pmiR-181a multipolar cytokinesis merge
